# 3D bioprinting and its innovative approach for biomedical applications

**DOI:** 10.1002/mco2.194

**Published:** 2022-12-24

**Authors:** Swikriti Tripathi, Subham Shekhar Mandal, Sudepta Bauri, Pralay Maiti

**Affiliations:** ^1^ School of Material Science and Technology Indian Institute of Technology (Banaras Hindu University) Varanasi India

**Keywords:** 3D bioprinting, bioprinting technology, cancer therapy, fabrication strategy, tissue engineering

## Abstract

3D bioprinting or additive manufacturing is an emerging innovative technology revolutionizing the field of biomedical applications by combining engineering, manufacturing, art, education, and medicine. This process involved incorporating the cells with biocompatible materials to design the required tissue or organ model in situ for various in vivo applications. Conventional 3D printing is involved in constructing the model without incorporating any living components, thereby limiting its use in several recent biological applications. However, this uses additional biological complexities, including material choice, cell types, and their growth and differentiation factors. This state‐of‐the‐art technology consciously summarizes different methods used in bioprinting and their importance and setbacks. It also elaborates on the concept of bioinks and their utility. Biomedical applications such as cancer therapy, tissue engineering, bone regeneration, and wound healing involving 3D printing have gained much attention in recent years. This article aims to provide a comprehensive review of all the aspects associated with 3D bioprinting, from material selection, technology, and fabrication to applications in the biomedical fields. Attempts have been made to highlight each element in detail, along with the associated available reports from recent literature. This review focuses on providing a single platform for cancer and tissue engineering applications associated with 3D bioprinting in the biomedical field.

## Graphical abstract

Design and discovery pipeline of 3D bioprinting technology for biomedical applications.

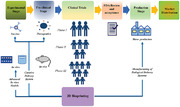

## 1 INTRODUCTION

Since the 15th century, printing has been known as one of the most vital processes of producing texts or images for quicker and broader information dissemination. It is also known as a novel and creative way to transfer information. It has also marked an impact on society by affecting the nation's education, politics, religion, and language.^1^ Since 2D printing is a high‐cost technology, increasing the time and reducing the scalability of developing a particular product is the need of the hour. These limitations are overcome by these 3D printing technologies as they helped overcome various manufacturing challenges globally.^2^, ^3^ In 1983, Chuck Hull invented stereo lithography (SL), also called 3D printing, thereby he is popularly gaining as the father of 3D printing.^4^ This novel approach globally transformed printing technology to a new level. It also proved to be a new door for the industries, manufacturing and medical technologies in making technological advancements by overcoming specific challenges. This invention modified the earlier 2D printing technologies and helped to advance from 2D to 3D components by using different additives that create a successive multilayer to form the desired 3D shapes.

**FIGURE 1:**
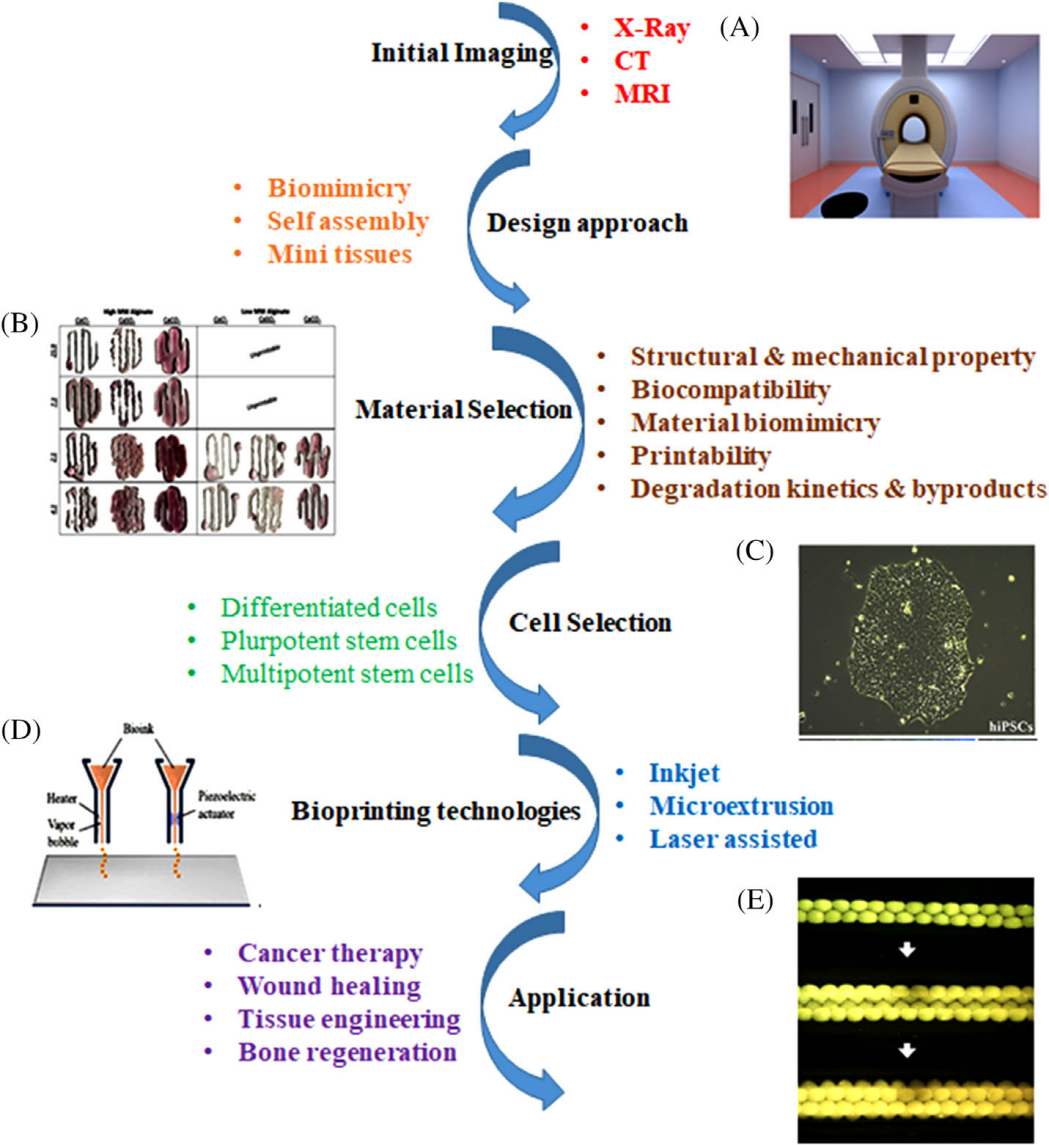
Step‐by‐step schematic processes involved in 3D bioprinting. (A) Magnetic resonance imaging (MRI) reproduced with permission from Ref. 6, copyright 2018 MDPI. (B) Printability of alginate for 3D printing of tissues reproduced with permission from Ref. 9, copyright 2017‐The Author(s). (C) Pluripotent cell reproduced with permission from Ref. 7, copyright 2021 The Author(s). (D) Inkjet Printing reproduced with permission from Ref. 10, copyright 2019 The Author(s). (E) Pattern of multicellular spheroids assembled into tubular form reproduced with permission from Ref. 8, copyright 2009 ‐Elsevier.

3D printing defines the layer‐by‐layer deposition of bioinks (tissue spheroids, microcarriers, cell pellets, etc.) in an exceptionally designed fashion as prescribed by a software‐supported system to create the desired 3D structure.^2^ Earlier, this technique was used only from the mold to develop the desired 3D structures from the biological materials. However, the designs became more complex upon developing the technology from resin‐based to solvent‐free aqueous system. The introduction of direct printing of biomaterials with or without the incorporation of live cells could further be used for transplantation. With rapid technological advancement in cell biology and material science, 3D bioprinting was better modernized, and tissues were incorporated into the complex models making the researcher close to eradicating the problem. These tissue engineering models have been used to create medical devices in prosthodontics.^1^ Although bioprinting is increasing, it has faced several problems in every aspect, from materials to incorporating live cells into the fabricated system. Biomaterial selection is one of the significant steps in fabricating the structure. The pure synthesized materials are functionalized and incorporated with many functionalities and associated groups, which increase the biocompatibility of materials as compared with pure materials. Several factors guide this transition. These factors include proper control over the mechanical properties (macro and microscale), achieving tissue designs with physiological heterogeneity, developing methods to extract and expand functional cells from stem cells, and interfacing the bioprinted tissues with a specific physiological vasculature network. Many models at the early stages lack essential elements like vasculature, lymphatics, and several practical and supportive cell types necessary for the normal functioning of large and complex tissues/organs. Due to these challenges, earlier models included the superficial cells and tissues, but later models had an advanced version with clinically relevant complex geometries.^5^ Mainly, 3D bioprinting comprises three main components: biomaterials, cells, and growth factors. The live cells are incorporated with the biomaterials and printed into a desired complex form with the help of various types of printers. These growth factors can also be functionalized to enhance biological and cellular activity making the system closer to the real human body models for several biomedical applications. Initial imaging needs to be done to construct a designated 3D bioprinted structure. Various diagnostic tools like MRI, CT, and X‐ray are popularly used these days for imaging.^6^ The diagnostic is followed by deciding the design, material, and cell selection, as shown in (Figure 1).^7^, ^8^, ^9^, ^10^ Upon careful selection of bioink, bioprinting technology plays a significant role in forming the complex construct to use in various applications. Conventional approaches involved thoroughly optimizing various parameters for batch manufacturing. In 3D printing, any prior formula or ingredient optimization is not affected. It deals with the incorporation of different sophisticated software that smoothens the processing and helps give the predictability of the desired result beforehand. It does not require prior optimization of the quality and quantity of desired biomaterials. Earlier development of these complex models involved high cost, time, effort, and resources with reasonably low chances of success on the first attempt. 3D printing provides a highly efficient, resourceful, and cost‐effective personalized approach through its highly advanced imaging and additive manufacturing techniques. These techniques allow fast design and development of specific complex models suitable for the desired disease, application or location instead of a population‐centric approach.^4^ The processes involved, materials used, and possible applications for 3D bioprinting are schematically shown in (Figure 1).

With increasing advancement, the market size of 3D bioprinting is valued at 1.7 billion USD alone in 2021 and is expected to reach 1.94 billion by 2025. This value is increasing and is expected to reach the compound annual growth rate (CAGR) of 15.8% from 2022 to 2030.^5^ Several reasons for this high growth include limited organ donors, rising R&D investment, technological advancement, and other private funding sources. Financial and technical assistance is provided across different development phases of the printing techniques. Switzerland‐based bioprinting company from the European Union in May 2021 has developed a miniature model of the pancreas using their Tomolite 3D bioprinting technique. The Curtin University was also supported by their Australian counterpart to develop and optimize the 3D technology for bioprinting skin tissue directly onto model wounds as a prologue to human skin restoration. Another Canadian‐based industry, Aspect Biosystems, also worked in this field to enhance its technology for the 3D printing of human tissues.^11^

The global pandemic has left several types of chronic respiratory diseases, which has helped in the sudden growth of the market size of 3D bioprinting. With every new COVID‐19 wave, new hurdles are faced by healthcare professionals, the community, and also the government as to how to reduce its effect and aftermath. The last few waves faced the lack of availability of test kits for COVID‐19. Several 3D bioprinting companies handled this major problem, and test kits were manufactured on a large scale. One of the U.S.‐based companies, Formlabs, reportedly manufactured 100,000 nasal swabs for COVID‐19 testing each day.^12^ Recently, many pharmaceutical companies, R&Ds, and healthcare workers are rallying to fight this deadly global pandemic in every possible way. Stratasys is one of the leading manufacturers of 3D printing in America and has manufactured face shields with the help of a 3D printer. Around 100,000 face shields were shipped in the US until March 2020.

The present review highlights the important parameters from developing several bioinks to the fabrication of the 3D printed constructs along with the ongoing and completed clinical trials. This review also focusses on the criteria for selecting biomaterial for the crosslinking strategy and its application in biomedical arena. It has been elaborated on the biomedical application by discussing its use in cancer models and tissue engineering. Further, this review also covers the perspective introduction of machine learning (ML) and 4D printing.

## 2 MAJOR APPROACHES FOR 3D BIOPRINTING

3D printing is one of the viable and most efficient approaches to the problems faced by 2D printing structures. Flexible design, rapid prototyping, print‐on‐demand, and strong and lightweight parts are the benefits of 3D printing over 2D printing. To print a complex structure, one needs a proper way or approach to overcome the specific issues and design the system accordingly. 3D bioprinting has been used to construct 2D tissues for solid organs. Skin, hollow tubes (blood vessels), hollow nontubular organs (bladder), and other solid organs such as kidneys can be constructed using 3D bioprinting. Hollow organs are more complex to construct as compared with solid ones. Thus, they take an unusually long time to develop.^1^, ^3^ Scientists have developed different methods to produce living components, structures, and organs with similar biological and mechanical properties. Here, three main central approaches are described, for example, biomimicry, autonomous self‐assembly and mini tissue building block.^1^

### 2.1 Biomimicry

Millions of years of evolution have molded the world around us and have led to the creation of vast amounts of incredible and magnificent things. Per the definition, biomimicry makes technological and industrial designs by copying natural processes/objects. The main idea behind biomimicry is to observe nature, learn the process, and try to solve the challenges already available in nature.^11^ It helps to create, fabricate, synthesize, or engineer structures identical or similar to the natural structures as observed. In biological structure, intracellular and extracellular components and the environment of tissues and organs in the human body are the essential factors while the synthesis and functioning of any 3D constructs. It needs to duplicate the shape, framework, and micro and macroenvironment of the organs and tissue of any human body. The 3D printing platforms enable the multimaterial printing of complex 3D constructs consisting of living cells and a vivid variety of biomaterials and growth factors. To construct these complex structures, one must have thorough knowledge and understanding of the microenvironment, structural arrangement, biological factors, and composition. Structural arrangement includes the organization of functional and supporting cell types. Growth factors include the gradient of soluble and insoluble factors. The constituent of the different cellular environments and the nature of the biological forces present in the microenvironment are essential for accurate design.^1^, ^5^, ^12^

Replicating biological tissues on the microscale level is an essential step for these complex structures. Gecko lizards are known for their sticky pads as they can walk up the smooth surface like stone walls and glass. The special microscopic hairs help present on their pads help them stick to walls vertically. Scientists have used this property to develop adhesives to create wounds without stitches.^13^ Tissue‐tissue and organ‐organ interfaces were created using organomimetic microdevices. A liver chip was formed using hepatocytes cells and flow chambers detached by a microfabricated baffle as a barrier. The barrier separated the cultured hepatocytes from the fluid flow to mimic the endothelial–hepatocyte interface of the liver sinusoid. The structure's geometry plays a significant role in separating the cell chamber, promoting the linear arrangement of hepatocytes in two lines, thereby facilitating the production of functional bile canaliculi along the hepatic‐cord‐like structures.^14^

In vitro model of tumor–stromal interactions was engineered in a microfluidic device. The device consists of two poly (dimethylsiloxane) (PDMS) microchannels separated by a semipermeable membrane. Cancer cells were deposited in a pattern in the top channel at a spatially defined position relative to the source and sink cells. This particular pattern was used to create physiological chemotactic gradients that helped the migration of the cancer cells. The alveolar–capillary interface was recreated in the breathing lunch‐on‐a‐chip system. The 3D architecture of the angiogenesis model was created using a microfluidic model. In this model of the human umbilical vein, endothelial cells were cultured in two parallel microchannels, separated by a 3D collagen gel. The sprouting in the endothelial system was optically monitored while applying fluid shear stress. It was also induced by interstitial flow through the 3D collagen gel (100 mm, bar).^14^ Researchers have taken inspiration from normal grass as they are super lightweight, but at the same time, they are very robust. The grass can bend when we step on it, but it returns to its natural shape due to its microstructure. Porous, cellular microstructure and its hollow microstructure are the main reasons for the attractive property of grass. Scientists used both these fascinating properties and came up with the idea of ceramic ink. They have helped to produce tissue scaffolds, thermal insulators, and lightweight structural materials. The ceramic foam developed by the scientist is made out of natural materials such as water, air, and alumina particles.^15^ The development of vast knowledge and research from engineering, imaging, biomaterials, cell biology, biophysics, and medicine is required for successfully creating these complex similar artificial models mimicking natural structures and functions.

The layer‐by‐layer fabrication requires high precision and repeatability as it is essential for completing the goal of imitating the tissue and cell‐specific composition of extra and intracellular components. Sometimes, the development of a more advanced bioprinting system acts as a catalyst in achieving the proposed biomimicry using various types of bioink in a single approach.^16^ Wanjun Liu and his research group used the extrusion‐based bioprinting technique to command the dispensing pattern through a single nozzle by utilizing up to seven controllable valves in a rapid and continuous fashion.^17^ This strategy increased specificity and functionality in the fabricated ECM components at a predefined spatial position. Bioink plays an important role in the fidelity and cell viability of the printed complex constructs. Several bioprinting strategies have been enhanced and implemented to manipulate the microenvironment needed for printing the 3D design. This manipulation can be achieved by controlling the reversible crosslinking mechanisms of composite polymer bioinks and by hybrid bioprinting of both cell‐laden hydrogels with synthetic biodegradable polymers of different volumes. Nanostructuring, macromolecular crowding, and reinforcing thermoplastic polymer can be used vividly to transform the microenvironment of the printed complex.^18^, ^19^, ^20^, ^21^ The composite hydrogel facilitated good printability for achieving good structural integrity. Muller and his research group used composite hydrogel by mixing acrylate with the unmodified Pluronic F127; it displayed an excellent printing property of pluronic and stable gel created using UV crosslinking. The system increased the cell viability from 62 to 86% on days 14.^18^ Pluronic F127 has also enhanced the efficiency of the crosslinked PEG‐fibrinogen conjugates in other studies.^19^, ^22^ Natural polymers like gelatin and alginate have also been incorporated into the hydrogel to form the composite, enhancing the printability and cellular viability of the printed constructs.^23^, ^24^, ^25^ Chitosan is also used for 3D printing technology because of its biocompatible, biodegradable and antimicrobial properties. However, when used alone, it has slow gelation and low mechanical properties. Gelatin is used to make hydrogel composite, thus, leading to better osteogenic cell proliferation and differentiation. Hence, good printability at room temperature, high 3D constructs shape fidelity, and good biocompatibility can be achieved.^26^, ^27^, ^28^ Macromolecular crowding has been used to describe intra and inter‐cell biochemistry. Collagen is used within most native tissues to form highly complex hierarchical structures within the native tissues.^29^, ^30^, ^31^ PCL is used as a reinforcing polymer due to its good biocompatibility, comparatively long degradation time, and low melting temperature. The rapid cooling property also avoids the damage caused to the cells due to the high temperature processing while constructing the 3D printing design.^32^ The graphene/PCL composites were used for neural tissue regeneration and promoted chondrogenic differentiation of MSCs in the scaffold fabricated using the SL‐based printing techniques.^33^

### 2.2 Autonomous self‐assembly

Self‐assembly is a process where atoms, molecules or nanoscale building elements spontaneously organize themselves into ordered structures or patterns with nanometer features without human intervention. It is one of the most promising practical, low‐cost, and high throughput approach for nanofabrication.^34^ This approach helps to replicate the tissues of interest using embryonic organ development models.^12^ Tissue structures present at earlier stages have different structural and biological components compared with the later stages. In the early development stage, the cells create their extracellular matrix (ECM) components, specific cell signaling, autonomous organization, and patterning. These properties help them to give specific biological functions and microarchitecture.^35^, ^36^ This approach needs information about the embryo's developmental techniques, including its tissues, organs, and functioning. The field of developmental biology has one of the best possible examples of tissue self‐organization and self‐assembly. One of the examples uses a “scaffold‐free” version of this approach. This version uses self‐assembling cellular spheroids with the property to undergo fusion and cellular organization to reconstruct the evolving tissues with the same structure and function. Researchers rely on the cells as the fundamental carrier of histogenesis, leading the tissues’ composition, localization, and functional and structural properties.^3^, ^37^ Autonomous self‐assembly is a complex phenomenon requiring information of the development phenomenon of embryonic tissue genesis and organogenesis. It also needs the ability to control the environment to initiate embryonic mechanisms in 3D bioprinted tissues. Okano et al.^38^ have developed sheet based approach for cardiac tissue engineering using a self‐assembly approach. Quick electrical coupling between the layered cardiomyocyte sheets was seen through functional gap junction formation after the harvest. Further, after the implantation in the subcutaneous position, pulsatile, layered cardiomyocyte sheets survived. The sheets developed for an extended period. The self‐assembly approach has been used for various cells, including the epidermal keratinocytes, kidney epithelial cells, and periodontal ligaments.^39^, ^40^, ^41^

### 2.3 Mini tissues

This approach is mainly the combination of both mimicry and self‐assembly approaches. It is relevant in both the strategies mentioned earlier.^42^ As the name says, mini tissues comprise smaller, functional building blocks of organs and tissues. For example, the nephron is the mini tissue for constructing kidney tissues. Two major strategies can be seen in this approach. First self‐assembling cell spheres are gathered into a macrotissue through biologically inspired design and organization.^42^, ^43^ In the second strategy, accurate high‐resolution reproductions of tissue units are designed, followed by their self‐assembling into a functional macrotissue. The emerging mini tissue‐based approach in tissue engineering has made a wide variety of improvements in the 3D printing of complex structures. This approach is generally based on the developmental biology‐inspired assumption that 3D materials of specific required material and composition could be fabricated without solid porous biodegradable synthetic or natural scaffolds. It also demands the synthesis of more sophisticated soft natural biomaterials and ECMs such as hydrogels.^44^ Mini tissues approach was used for the Self‐assembly of vascular building blocks to make a branched vascular network.^45^, ^46^ “Organ‐on‐a‐chip” is constructed using functional tissue units and sustained and associated by a microfluidic network. It is used in in vitro models of disease for the screening of drugs and vaccines.^47^, ^48^ Gu et al.^49^ created neural tissues by printing human neural stem cells, differentiating in situ into functional neurons and supporting neuroglia. Polysaccharide‐based bioink using alginate, carboxymethyl‐chitosan, and agarose were used as a biomaterial for encapsulating the stem cells for in situ expansion and differentiation. The differentiated neurons formed synaptic contacts and established networks. Spontaneous activities were seen in the neurons, and as a result, calcium response increased, and gamma‐aminobutyric acid expression was predominant. Axel Gunther and coworkers used a microfluidic device to fabricate a resistance artery structure and function under physiological conditions under 37°C and 45 mm Hg transmural pressure. This device allowed on‐chip fixation long‐term culture and fully automated acquisition of up to ten dose–response sequences of complete mouse mesenteric artery segments in a definite environment having 250 μm diameter and 1.5 mm length. The phenylephrine or acetylcholine application caused dose–response relationships, which were virtually similar to the conventional myography.^50^

The above strategies have been used in several bioprinting approaches for creating a 3D printed construct for the desired functional, mechanical, or structural property. These strategies can produce a construct that can produce multiple components and properties simultaneously. Material selection is one of the crucial steps in fabricating the 3D printed system. These systems are used for in vitro analyses after successful and desired in vivo fabrication.

## 3 MATERIALS SELECTION CRITERIA

Printing technology was mainly related to nonbiological applications like firearms, military, and certain manufacturing products. These applications mainly deal with organic solvents, high temperatures, and other crosslinking agents, which help deposit metal, ceramics, and thermoplastic polymers. These processing conditions are not suitable for biological materials and live cells. Material selection is essential for printing desired complex biological models with specific mechanical and physical properties to fulfil the desired applications. As mentioned earlier, materials used for the 3D printing techniques have been denoted with a particular name of bioinks. This printing method manufactures a wide range of complex structures using ceramics, metals, and polymers and their combinations in various hybrids to form composites. Materials used for biomedical applications are mainly natural or synthetic polymers. Natural polymers are primarily similar to the human ECM and have natural bioactivity, making the models closer to the original shape. Naturally found polymers include alginate, gelatin, collagen, chitosan, silk, HA, fibrinogen, agar, and other biocompatible polymers used alone or incorporated with other polymers to form a suitable matrix.^51^ These polymers include the main component of bioinks. At the same time, synthetic polymers like acrylonitrile butadiene styrene (ABS), poly(lactic acid) (PLA), poly(glycolic acid) (PGA), poly(lactide‐co‐glycolic acid) (PLGA), polyurethane (PU), polyamides, and several other polymeric hydrogels can be functionalized and molded with specific properties to match specifically designed applications.^52^, ^53^, ^54^ Although poor biocompatibility, toxic degradation products, and loss of mechanical properties during degradation are some disadvantages of synthetic polymers, they are still one of the primary materials used for synthesis.

Materials used for this application should have long‐term and short‐term stability as they are desired to be incorporated with the cells. Long‐term stability mainly includes biocompatibility as it is a long process. The desired bioink needs to remain biocompatible at every fabricated stage until the desired stage has been achieved. Short‐term stability is necessary to maintain the integrity of the material at the initial stage by ensuring correct tissue structures such as pores, channels and networks and by keeping that they do not fail until printing has been done. Bioinks should have different structural and printing properties to be considered an ideal material for fabricating complex 3D structures using 3D Bioprinting. An ideal bioink should have the desired physiochemical properties like mechanical, biological, rheological and chemical characteristics. These properties lead to (a) fabrication of tissue constructs with desired mechanical stability and robustness along with retention in the tissue matching mechanics, mainly in a tunable matter, (b) gelation and stabilization should be adjustable to help the bioprinting of structures with high shape reliability, (c) biocompatibility and biomimicry of the natural microenvironment of the tissue, (d) suitable for chemical modifications to get desired tissue environment, (e) potential to have large scale production with minimum batch‐to‐batch variations.^55^, ^56^, ^57^ The material's printability, biocompatibility, degradation kinetics, byproducts, structural, mechanical property, and material biomimicry are vital requirements for 3D bioprinting.

### 3.1 Printability

As the name suggests, it includes the property of the material that makes it easy and most suitable to print to form the desired shape. It is essential as it allows the material to be deposited precisely while maintaining the 3D control. The printing capability of a material is one of the limiting factors present in different types of printers for printing complex 3D constructs. Inkjet‐type printers have limitations on the viscosity of materials, but in microextrusion, materials require specific crosslinking mechanisms or shear thinning properties. Parameters like time and nozzle gauge also affect the printing process, and in turn, it affects the quality of printed 3D material.^58^ The nozzles and/or energy used to ejaculate the bioink is generally limited by the factor of viscosity of liquid and surface tension.^59^ Crosslinking of polymers is also a vital phenomenon for printing 3D constructs. Specific crosslinkers are required for inkjet printing as they help in layer‐by‐layer formation for 3D structures. However, in the case of microextrusion, final crosslinking is done after fabrication to incorporate the deposited highly viscous material.^2^ Cell viability defines the number of healthy cells present in a sample. It is an essential indicator of the proliferation of cells for understanding the mechanism in action of specific bioactive components like genes, proteins and other biomaterials.

Crosslinking is an essential part of the formation of complex 3D constructs. It allows the formation of the bonds between the materials and thus acts like a substrate for cell incorporation. It also helps to provide a structural framework for the printed system. Various crosslinking methods are used for 3D printing, for example, polymer crosslinking, photo crosslinking, and thermal crosslinking, and so on.^43^, ^60^ The viscosity and flow rate of the materials also affect the printing and cellular study phenomenon. Biological materials with either low thermal conductivity or the capacity to cushion cells during delivery may enhance cell viability and function in the case of thermal inkjet printing and laser‐assisted printing (LAB).^61^, ^62^ However, printing the cell viability may depend on the printer specifications, material properties, resolution, and the cell types used to print with the material. Out of inkjet, microextrusion and LAB, only LAB and inkjet printing have viability greater than 85%, whereas microextrusion printing gives cell viability between 40 and 80%.^63^, ^64^, ^65^

### 3.2 Biocompatibility

It is one of the most common terminological requirements in biomedical applications. It is considered one of the primary and the most vital features of a material to be suitable enough to be used for a biological or biomedical application. Biocompatibility can be defined in many ways, but all the definitions reach one robust understanding: the material should be compatible with the living cells, tissues or organs so that it can be incorporated together, printed and ultimately form a complex 3D model. The material should cohabit with the host's internal tissues without causing any unwanted local or global effect. Several factors can be considered for biocompatibility. The material should not cause any inflammatory or immune response, should be biodegradable and has similar function and behavior in situ and ex situ. The material should be versatile enough to be functionalized so that it can be used to enhance the efficiency of any desired application. The byproducts formed upon fabrication or disintegration should not be less or no harmful to the host. The materials should also facilitate proper cellular, mechanical and molecular signaling systems for the host's essential functioning, especially in organ transplantation.^1^, ^64^ Many natural and synthetic materials are used for 3D printing for biomedical applications. PLA is extensively used for biomedical applications such as breast reconstruction surgery.^66^

### 3.3 Degradation kinetics and byproducts

One of the key mottos of 3D printing is the reconstruction of the desired organs or tissues. It also helps to print or design scaffolds, which can be used to deliver biological components like drugs, DNAs, and proteins in a controlled manner. Upon delivery, these scaffolds must be degraded to cause the least or no harm to the host. When the scaffolds devalue, the incorporated cells secrete proteases and produce ECM proteins that outline the new tissues.^67^ Many factors are considered during the kinetics of degradation. Different enzymes can degrade each polymer in different reaction environments (Table 1). The degradation rates should match the cell replacement ability of materials with their own ECM proteins. The degradation by products should be nontoxic, readily metabolized, and instantaneously excreted from the body of the host. Upon degradation of any polymer, it clears to small molecular weight polymers that can be recognized by other cells. It may cause inflammation and other deteriorating effects on the host. Sometimes few polymers swell and contract to cause inhibition in the proper functioning of cells with the materials. The extra fluids in the surroundings are sometimes absorbed by the polymers causing them to swell. In other cases, the contraction of the polymer causes variation in the size and weight, sometimes closing pores or vessels and impacting the migration and cell delivery process. Degradation products are sometimes used to define any polymer's biocompatibility at its preliminary stage.

**TABLE 1:** Various bioink polymers used in 3D bioprinting

Bioink polymers	Cell viability	Gelation method	Mechanical property	Degradation property	References
Agarose and its blends	>70%	Thermal/ionic	Better mechanical strength	Protease XIV enzyme caused a 45% reduction in mass after 28 days.	^68^, ^69^, ^70^
Alginate	90.8%	Ionic crosslinking	Pure alginate has low mechanical stability	Good degradability	^71^, ^72^, ^73^
Gelatin	91%	Thermal	Mechanical strength increases with crosslinking	More than 90% degraded after 35 days in the presence of l‐lysine diisocyanate ethyl ester	^74^, ^75^, ^76^
Hyaluronic acid with gelatin	>95%	Photoinitiated gelation	–	Enzymatic degradation at 37°C, controllable degradation	^77^,^64^,^78^,^79^
Fibrin	74.27%	Fibrinogen‐thrombin	Unique viscoelastic properties among polymers	Controlled and adjustable biodegradation	^80^
Pluronic F127	91.3%	Photo‐polymerization	High elongation at break	More than 85% degradation in PBS after 1 week	^81^
Pluronic F127/alginate	85%	Ionic	Low mechanical property	–	^72^, ^79^
Gelatin methacrylate	75–90%	Photo‐polymerization	Mechanically strong	No significant degradation over the 2 months assay	^82^

### 3.4 Structural and mechanical properties

Maintaining the structural and mechanical integrity of the printed model is one of the crucial parameters for material selection. The materials should have similar mechanical properties like elasticity and strength to the native biological cellular and tissue components. These properties make the materials better fit than the replica of the original tissues. Mechanical properties vary depending on the different structural requirements from the outer skin to the innermost bone. Nowadays, sacrificial materials are used, which provide required structural and mechanical properties only for a certain period. These materials sacrifice themselves after the task has been over. They are either used at the time of printing for sufficient crosslinking for the complex model or incorporated into the model so that they can function efficiently until the assigned material can efficiently carry out the same function.^43^, ^60^, ^61^ Carbohydrate glass/elastomer was used as a sacrificial material for the 3D printing the soft elastomer.^83^ These sacrificial materials also affect the biocompatibility and degradation rate of the bioinks and can affect the host. Mechanical properties like tensile strength and stiffness also play a vital role in reconstructing and 3D printing bones.^84^

### 3.5 Material biomimicry

As described earlier, bio stands for biomimicry's life or natural components, and mimesis means imitation or resemblance. It explains the resemblance of the printed 3D constructs, which resemble biological components present in nature in an analogous or homologous way. It helps us observe and learn from nature to incorporate its patterns to find a solution for our designs. Biomimicry is one of the essential properties of 3D bioprinting. It helps to study the constructed complex natural systems inside and outside our biological system. It helps to fabricate identical cellular and an extracellular component of a tissue or organ.^61^ For fabricating a branching pattern of nerve cells, one needs to mimic the branching patterns of the vascular tree with physiologically correct biomaterial types and gradients.^3^ A thorough understanding of the micro and macroenvironment and proper composition of the specific material suited for that cause is required to understand the arrangement and position accurately.

The precise anatomical and architectural information helps to fabricate a precise 3D printed model. Various bioprinting techniques have been designed and improved for 3D construction. Different bioprinting techniques also affect the tissue and organ design of the construct. Some techniques form continuous structures, whereas discrete structures can be formed in some cases. The design and capabilities of the printed construct are very much influenced by the types and properties of the bioprinting systems and will be discussed in the next section.

## 4 BIOPRINTING TECHNIQUES FOR 3D PRINTING

**FIGURE 2:**
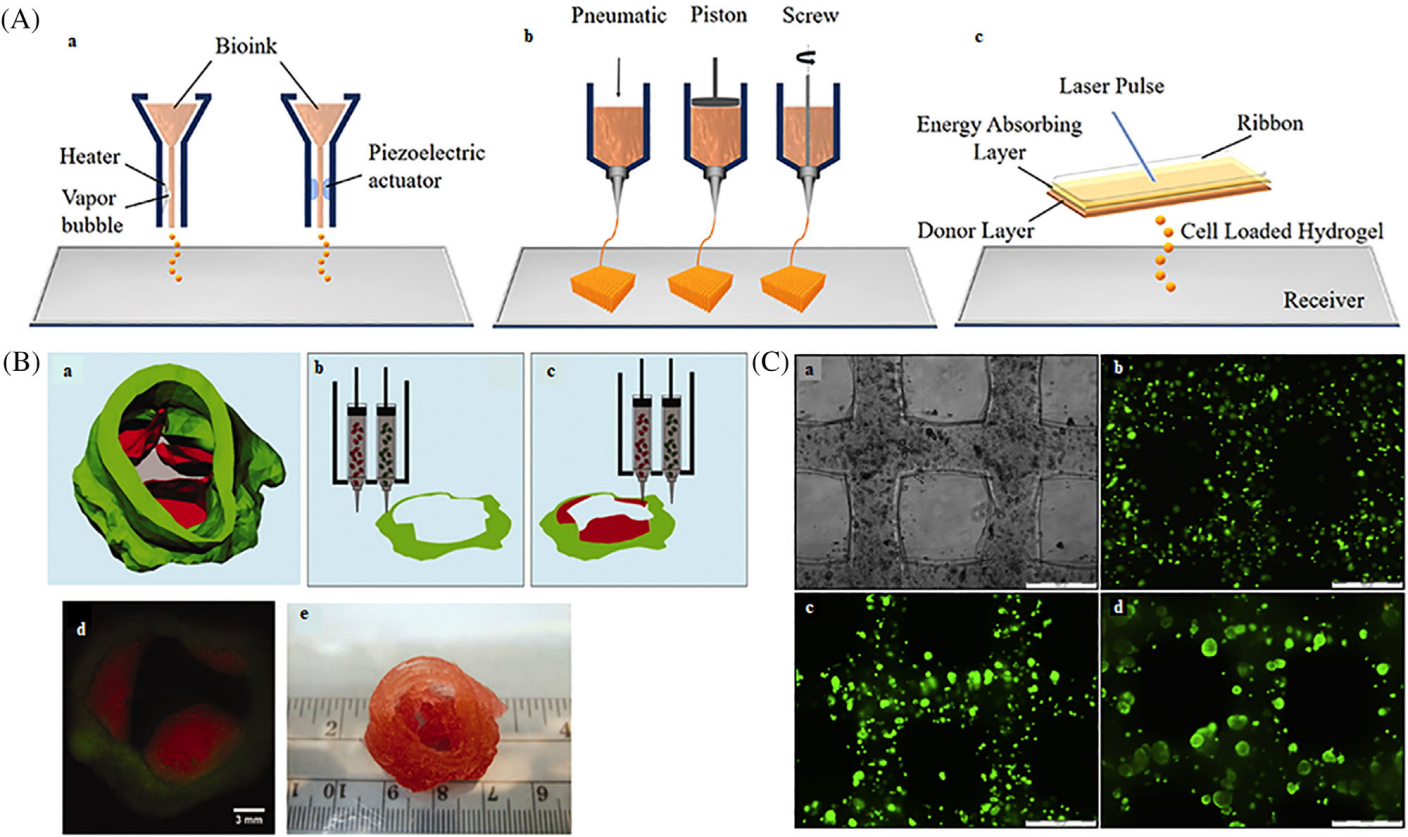
Various bioprinting technologies and printability. (A) Schematic presentation of (a) inkjet printing, (b) extrusion printing, and (c) laser‐assisted reproduced with permission from Ref. 10, copyright 2019 The Author(s). (B) (a) Aortic valve model reconstructed from micro‐CT images, (b) and (c) schematic illustration of the bioprinting process with dual cell types and dual syringes, (d) fluorescent image of first printed two layers of aortic valve conduct SMC for valve root were labeled by cell tracker green and VIC for valve leaflet was labeled by cell tracker red, (e) as‐printed aortic valve conduit reproduced with permission from Ref. 76, copyright 2013 ‐John Wiley & Sons, Inc.. (C) High viability cell‐laden gelatin scaffolds, (a) bright‐field images, cell viability within scaffolds in (b) Day 1, (c) Day 7, (d) Day 14 (scale bar‐500 μm) reproduced with permission from Ref. 181, copyright 2014 ‐Elsevier.

Technology is the term used to create systems or setups for any applications. It enables to use the scientific knowledge for any practical purpose. Bioprinting technologies enable printing by accurately depositing cells in the biomaterial in a specific orientation to form a complex structure using a computer‐aided printer. Factors like surface resolution, cell viability and the nature of biomaterials affect the type of technology used for 3D bioprinting. This process requires a medium to be suitable enough for the cells to acclimatize and survive to the printing system and biomaterials associated with it. Three major 3D printing technologies are used for various applications; Inkjet, laser‐assisted and microextrusion (Figure 2A).^1^, ^10^

### 4.1 Inkjet bioprinting

The inkjet printer was primarily invented by Ichiro Endo in Japan during the 1970s.^85^ Unlike dot matrix printers, it involved using many tiny dots of ink so small that naked eyes could not see them. In 1988, Klebe described the method of cytoscribing.^86^ This involves the cell being deposited over a substrate using cell adhesion proteins using a computer. It enables the establishment of precise spatial interrelationships between cells. It was deposited either by an inkjet printer or a graphics plotter. This method helped create a 2D patterned tissue onto a flexible substrate.^87^ After this approach, the inkjet printers were modified, and elevating the platform was introduced to provide a vertical movement. The inks were replaced by biological components, for example, cells, ECM components, and several other biological materials, leading to the use of inkjet printers for 3D bioprinting.^1^, ^43^, ^88^, ^89^ It is the second most common technology and is involved in most extrusion‐based bioprinters for commercial manufacturing. The recent trend shows a significant rise in the number of published papers per year from 2000 to 2020.^3^ These printers have 17% of their share compared with all other bioprinting techniques used in the market.^3^ Bioinks like alginate, PEG, fibrinogen/fibrin, hydroxyapatite, growth‐based bioink, PCL, PVP, and commercial bioink like Derma‐matrix are few of the materials used for the inkjet bioprinting technique.^57^ Inkjet printing is a noncontact printing technique that delivers a controlled amount of liquid solution to the specified location. Thermal or sound was used as a driving force for the ejection of the droplets onto the substrate.^63^, ^90^, ^91^, ^92^ It consists of thermal, piezoelectric, and electrostatic inkjet nozzles for printing cells and tissue scaffolds. Few modifications depend on the materials for deposition and the size of the complex model to be printed.^93^

#### 4.1.1 Thermal inkjet printers

As the name suggests, the electrical component is used to heat the solution and deposit it on the substrate. It is used to release an air bubble to break liquid into droplets.^89^ Thermal inkjet printers function by electrically heating the print head to produce pulses of pressure that force the droplets from the nozzle.^94^ The thermal element can heat up to 300°C. The high temperature is usually for the short term, causing the overall rise of 4–10°C; therefore, the biological components are unaffected by such a range of temperatures.^50^, ^96^, ^97^, ^98^, ^99^ The wide availability, low cost, and high print speed are the most importance advantages of this type of printing. However, the exposure to thermal conditions, mechanical stress, low droplet directionality, nonuniform droplet size, and frequent nozzle clogging tend to be disadvantageous here and reduce the efficiency of the printer (Table 2).^1^, ^3^, ^43^ The thermal inkjet printer was used to print hamster ovary cells and rat primary embryonic motor neurons.^34^, ^96^, ^100^ The fibroblast growth factor‐2 (FGF), ciliary neurotrophic factor (CNTF), and fetal bovine serum (FBS) was printed using a modified thermal inkjet printer (Canon BJC‐2100) on a polyacrylamide‐based hydrogel.^63^, ^101^ Thermal inkjet bioprinting was also used to fabricate neuroglia‐FGF‐2, CNTF, and FBS in a polyacrylamide‐based hydrogel. The printed substrate was further printed, and NSCs 38 cells were seeded on them. This printing was also used to print rat embryonic motor neurons and primary rat embryonic hippocampal and cortical neurons. These printed systems displayed greater viability.^63^, ^64^, ^89^

**TABLE 2:** Comparison of different bioprinting technologies^1^, ^64^, ^87^

Bioprinting techniques	Main components	Droplet speed	Viscosities	Resolution	Preparation time	Advantages	Disadvantages
Inkjet based	Polymeric liquid droplets deposited on solid substrate. Droplets should solidify before the next layer is ejected.	Size: 1–300 pl Speed: 1–10,000 droplets per second	3.5–12 mPa/s	<5–50 μm	Less	Easy modification Simple operation Good precision Fast printing speed. Color printing and readily available Nonflat surfaces can be used as substrates. Concentration can be varied.	The size is enormous. Limited variety of bioink Thermal and acoustic stress is generated on cells. A low viscous solution is needed due to the Clogging of nozzles Finite printing height. Lack of effective structural integrity.
Laser‐assisted	The laser pulse is used for heating biomaterial for deposition. Nozzle‐free, high précised printing occurs.	Speed: 200–1600 mm/s	1–300 mPa/s	>50 micron	Medium to high	A wide range of viscosity can be printed Cell viability is high Noncontact nozzle‐free printing. Nanoscale precision. Multiple cell deposition at once. High cell density bioink.	Limited control over printing direction. Difficulty in handling heterogeneous cells. UV lights may damage the structure. Limited photo cross‐linking agents can be used. The metallic residual can be found Tissue damage may occur due to laser light.
Extrusion	Nozzle/syringe is required for material deposition. High, medium, and low temperature can be achieved.	Speed: 10–50 μm/s	30 mPa/s–>6 × 10^7^ mPa/s	>5 micron	Low to medium	Wide range of biomaterial can be selected for printing. Largely open access software and hardware. Multiple material can be printed simultaneously Large organs can be constructed conveniently. Porous complex models can be printed. Ejection speed can be controlled.	Mechanical or shear stress causes low cell viability. Wide range of material having shear thinning ability can be selected. Gelation and solidification of the polymer is required. Pressure is generally increased causing low cell viability. Sometimes high temperature is not good for cells.

* Inkjet printer uses piezoelectric or electrical components for the source, whereas laser beam and heat are used as a source for laser and microextrusion printing, respectively.

#### 4.1.2 Acoustic inkjet

These printers use piezoelectric crystals to form the droplet at regular intervals and further deposit on onto the substrate.^1^ These crystals generate an acoustic wave inside the head of the printer. Piezoelectric crystals contain piezoelectric materials that undergo deformation when an external voltage is applied to system.^3^ These acoustic waves generated in the head of the system cause the liquid to break into droplets, which causes the easy ejection of the liquid from the nozzles. These waves can be adjusted to control the size of droplets as well as the ejection rates. These printers can generate and control uniform droplet sizes. Directionality is maintained in the ejection of the liquid and avoids the exposure of heat and pressure to cells, unlike thermal inkjet bioprinters.^87^, ^88^ Earlier single nozzles were used to eject and deposit a single type of material on the substrate and form 3D bioprinted models. As technology advances, multiple acoustic ejectors can be used simultaneously in an adjustable array format. These multiarray systems enable simultaneous printing of more than one kind of cells and materials on a similar substrate, thus, reducing the processing time.^89^ The piezoelectric materials require an external pressure or stimulus to produce the desired electric effect. These printers generally use 15–25 kHz frequencies to form the droplet, which can induce damage to the cell membrane and cause lysis of cells to be printed.^98^ The printer uses different types of materials; thus, viscosity of the systems varies in each case. However, the viscosity of the liquid affects the ejection of the liquid. Therefore, this mechanism limits the use of highly concentrated and viscous bioinks as their viscosity might dampen the applied acoustic waves and thus, they can obstruct the ejection of droplets. Generally, the viscosity of the material is kept below 10 centipoises as excessive force is required for high viscous material to eject droplets on to the substrate.^102^ Low viscosity solutions can avoid this, but it becomes inconvenient for printing 3D structures.^103^ A piezoelectric printing system was used to print bone morphogenic protein‐2 onto fibrin‐coated glass slides. This area was further used to culture the muscle‐derived stem cells isolated from adult mice.^89^, ^101^ Piezoelectric‐based systems were also used to print heparin‐binding epidermal growth factor (EGF) like macromolecules. The proliferation and migration of the external growth factor gradients were used for the in vitro study of mesenchymal cells.^104^, ^105^ Inkjet printing was used to fabricate full‐thickness skin models with pigmentation.^106^ After 1 day of culturing in the fibroblast medium, keratinocytes were printed on top of the dermal model and again placed in the culture medium for another day. This skin construct had individual and distinguishable epidermal and dermal skin layers. Freckle‐like pigmentation was seen on the skin construct upon maturation.^106^ The wound healing capacity of the 3D bioprinted skin grafts from inkjet printing has been studied.^107^ It has also been used to construct layered cartilage constructs in vitro through electrospinning (ES).^108^ Ease of modification, low cost, simple operation, fast color printing speed, and compatibility with many biological materials are the advantages associated with this type of printing. It exhibits a high resolution of 5–50 μm and high cell viability.^3^ This process provides the accurate position of droplet deposition on the substrate as well as simultaneous deposition of multiple cell types, as mentioned previously.^108^, ^109^ This printer allows electronic control over the droplet size and deposition rate and can vary from 1 to 300 μl volume with the rate of 1–10,000 droplets per second (Table 2).^46^, ^89^, ^95^ Drop sizes have been altered in various patterns by introducing concentration gradient of cells, materials or growth factors.^98^, ^99^ Even a single drop can be deposited having only one or two cells in line with ∼50 μm width.^109^ However, this process has certain limitations that restrict its use for fabricating all types of 3D bioprinted constructs. The low polymer viscosity, bulky size, low cell density (less than 10 million cells/ml) and low structural heights are the few limitations of this type of printing.^98^ Higher viscous materials cause clogging in the nozzles and reduce shear stress. In the case of low viscosities, crosslinking agents are often used, but sometimes they cause reduced printing processes and change the chemical and structural properties of materials.^5^, ^101^, ^110^ Further, some crosslinking agents require specific products or conditions toxic to cells causing reduced cell viability and functionality of the printed constructs.^111^

### 4.2 Extrusion bioprinting

Extrusion is a technique where the molten polymer is forced through a die to produce components of fixed cross‐sectional areas to produce rods, sheets, pipes, films, wire insulation coating, and so on. The material is conveyed forward by a feeding screw and is forced through a die, thus forming into a continuous polymer product.^112^, ^113^ These are the most common and inexpensive nonbiological 3D printers. It is a widespread bioprinter due to its low cost and ease of use.^1^ This type of printing was first introduced in 2001, but it expanded primarily after 2015 upon expanding commercial bioprinters in the market.^34^, ^93^ It consists of 39% of the total shares owned by bioprinters in the international market.^3^ The low‐cost technology allows extrusion‐based bioprinters to be the most studied printers in the literature and allows printing a wide range of materials.^114^ The high demand for extrusion‐based printers has allowed a significant increase in papers from 2000 to 2020.^3^ Roughly 30,000 printers are sold worldwide every year, and among them, academic institutions have mostly preferred microextrusion technology for research in tissue and organ engineering.^1^ Microextrusion printers are used primarily for industrial purposes. They tend to be more expensive due to advanced features like better resolution, spatial control, speed, and versatility in the material to be printed.^1^, ^102^, ^103^, ^104^

Extrusion‐based bioprinting is a pressure‐driven approach in which bioink (polymer solutions with or without cells, growth factors and other bioactive components) is extruded through a nozzle. The ejection or extrusion is pneumatic or mechanically assisted, and the droplets are deposited on the substrate in a predesigned manner.^3^, ^103^ This type of printer consists of several components. A temperature‐controlled material handling and dispensing system and a stage are present, with one or both capable of moving along the *x*, *y*, and *z* axes. It contains a fiber optic light source, which illuminates the deposition area and can act as a photo initiator. The printer is in control through CAD‐CAM software, and the images are recorded using a video camera capable of having movement in all three directions. Few systems consist of multiple print heads that enable the serial dispensing of various materials simultaneously.^37^, ^103^ The polymeric solutions are solidified using chemical and physical processes, including sol–gel transformation, polymerization, crosslinking, and various enzymatic degradations.^115^, ^116^ Materials whose viscosity lie in the range from 30 to 6 × 10^7^ mPa/s and are more compatible with these types of the printer as higher viscosity materials provide structural support for the printed model, and lower viscous material may provide a suitable environment to maintain cell viability and other functions.^117^ These printers include pneumatic or mechanical dispensing systems for the ejection of liquid. Mechanical dispensing systems deliver more direct control over the flow of material as delay of compressed gas volume is seen in these systems. Mechanical systems have smaller and more complex components, but pneumatic printers have simpler drive‐mechanism components.^113^ Screw‐based systems are more beneficial for distributing hydrogels with higher viscosities as they give more spatial control. Sometimes pneumatic systems are suited for distributing high viscosity materials.^65^, ^118^, ^119^, ^120^, ^121^ Microextrusion printing technology uses a wide selection of materials and porous constructs as it can deposit dense cells very highly. It helps to control ejection speed, multimaterial printing, and high resolution and is convenient to fabricate large organs compared with other printing technologies.^1^ Multicellular cell spheroids were deposited and allowed to self‐assemble into the desired 3D construct. These spheroids act as a homologous and anatomical system to the ECM tissue.^8^, ^43^, ^44^ These self‐assembly structures can possibly accelerate and direct the formation of complex structures. The mechanical microextrusion printing approach was used to fabricate scaffold‐less tissue spheroid. This printing method has several advantages, but they are not so efficient in constructing complex 3D structures. Cell viability is one of the most important parameters in biomedical applications. Microextrusion printing has lower cell viability as compared with inkjet printing. The cell survival rate of 40–86% decreases with increasing extrusion pressure and nozzle gauge (Table 2).^65^, ^122^ Factors like temperature and nozzle size can affect the cell viability of the printed system. These parameters are important for the researchers to present essential functions of printed 3D tissue constructs. Cell viability can be retained using low‐pressure conditions, and nozzle size can be increased in some cases, but these changes may cause major loss in the resolution and print speed.^58^ Limited material selection is another problem faced by this type of printer. The use of improved biocompatible materials might help to maintain cell viability and function after printing. Crosslinked hydrogels are mechanically strong, and sometimes they develop secondary mechanical properties that help increase cell viability and cause sturdiness in printed 3D constructs.^110^, ^123^

Moreover, improvements in the nozzle, syringe, and motor control systems reduce print times and allow the simultaneous deposition of multiple types of materials.^124^, ^125^ The extrusion technique requires materials with high water content and the ability to suspend cells; hydrogels are primarily used for the technique. Natural materials like gelatin, alginate, agarose, chitosan, dextran, fibrinogen, gellan gums, HA, and so on are used. Poly(ethylene glycol)s (PEG), pluronics, polyacrylamide, and poly(2‐hydroxyethyl methacrylate) are the synthetic polymers used for this technique.^126^, ^127^, ^128^

This technique was used to regenerate an ear formed of auricular cartilage and fat tissues. The hydrogel of PCL and PEG was used along with the chondrocytes and adipose‐derived stromal cells to fabricate an ear‐shaped scaffold. The system's viability was found to be more than 95%.^129^ In another system, similar materials were used to fabricate cartilage scaffolds by extruding alginate hydrogel onto PCL. The chondrocyte printed scaffold system has 85% viability toward the cells. Both with and without turbinate‐tissue‐derived mesenchymal cells scaffold were printed within the alginate bioink. The cellular printed system caused an increase in ECM production without any severe effect upon implantation into the dorsal subcutaneous spaces of mice.^130^ A hierarchical cell‐laden structure was fabricated to mimic multicellular tissues. In vitro studies were performed using PEG diacrylate and methacrylate gelatin (GelMA) hydrogel. The hydrogel was incorporated with NIH/3T3 fibroblasts and C2C12 skeletal muscle cells to print structures, which mimic the musculoskeletal junctions, muscle strips and tumor angiogenesis. Proper proliferation rate and interfaces were seen in the system after 3, 5, and 7 days of cell culture.^131^ Biodegradable conductive polymers like tetraaniline‐*b*‐polycaprolactone‐*b*‐tetraaniline were synthesized, and 3D printed using the extrusion technique to construct a porous scaffold for tissue regeneration.^132^ Pati et al.^32^ fabricated a hybrid scaffold combining PCL and decellularized ECM. The bioink was mixed with stem cells derived from adipose, cartilage, and heart tissues and deposited onto the PCL framework. The cell viability of the system was found to be 90% on day 7, and intracellular interconnectivity was observed within 24 h.^32^ Stimuli‐responsive conductive nanocomposite hydrogel was prepared using the extrusion technique. The hydrogel displayed good electrical conductivity, rapid self‐healing and adhesive properties, flexible and stretchable mechanical properties, as it is highly sensitive to near‐infrared light and temperature.^133^

### 4.3 Laser‐assisted bioprinting

LASER is an acronym for light amplification by stimulated emission of radiation. It is a device that monochromatic light through a process of optical amplification based on the stimulated emission of electromagnetic radiation.^134^ The laser was first built by Theodore H Maiman at Hughes research laboratories in 1960 by Charles Hard Townes and Arthur Leonard Schawlow.^135^ Laser‐based bioprinting uses laser‐induced forward transfer phenomenon to deposit a very small amount of bioink in liquid or solid phase.^87^, ^136^ It was initially developed to deposit metals onto receiver sheets, but it has been successfully applied in biological applications as well. DNA, cells, tissue, and organ printing are the typical applications where laser‐assisted printing is used.^96^ Odde and Renn developed this technique to print viable embryonic chick spinal cord cells.^137^ It has three parts donor side or ribbon, a laser pulse and a receiver side. The ribbon consists of a layer of transparent glass, a thin layer of metal and a layer of bioink. The transport ribbon acts as a support for the energy‐absorbing layer. The bioink is in liquid/gel condition and is transported from the donor side onto the receiver slide when the metal layer under the hydrogel is vaporized by a laser pulse.^138^, ^139^ All the components of this technique (laser energy, laser frequency, and biomaterial viscosity) have the potential to impact the resolution of printed complex structures.^140^ Surface tension, wettability of the substrate, the air gap between the ribbon and the substrate, thickness and viscosity of the biological layers affect the resolution of the printed construct.^138^ This technique is nozzle free, making it an excellent approach for depositing cells or materials, which cause clogging at nozzles. It can deposit cells at a density of up to 10^8^ cells/ml with a microscale resolution of a single cell per drop while using a laser pulse rate of 5 kHz with the speed of 1600 mm/s.^59^ It can print up to bioinks having viscosity up to 300 mPa/s.^141^ High energy laser pulse has a minimal effect on the cell's viability, structure, and function. It can cause selective writing of multiple cell types, but UV light may affect the cells.^142^ Laser‐assisted bioprinting has a comparatively low overall flow rate due to the rapid gelation kinetic required for high shape reliability.^143^

The formation of a ribbon is one of the crucial phenomena in printing complex structures. The preparation of individual ribbons is time consuming and causes difficulty during the codeposition of multiple biomaterials and cells. This simultaneous deposition can be time consuming since it is difficult to accurately target cells and materials to their desired location on the printing substrate. To overcome this difficulty “aim and shoot” procedure is developed. This cell‐recognition scanning technology is used, which enables the laser beam to select a single cell per pulse specifically, and it ensures that each printed droplet contains a predefined number of cells.^2^ Metallic residues are present in the final printed constructs due to the vaporization of the metallic layer from laser radiation. To avoid this phenomenon, a nonmetallic absorbing layer is used nowadays, and the printing process is modified such that it does not need an absorbable layer.^144^, ^145^ This system's cost is higher than other printing technologies due to monochromatic high‐energy laser beams. Owing to such disadvantages, laser‐assisted techniques for the printing of 3D complexes are less than 4% share of the total bioprinter available in the market.^114^ Bioinks like alginate, collagen, fibrin/fibrinogen, hydroxyapatite, matrigel and blood plasma are the few materials used for laser‐assisted bioprinting.^57^ This technique was used to fabricate a 3D printed skin model with specific cell densities in a layered tissue construct.^146^ Gruene et al. used laser‐assisted technology to print the biomaterial incorporated with the mesenchymal stem cells (MSCs). The study showed no effect of the laser pulse on the cells as no change in the gene expression occurred due to heat shock produced by the laser pulse. The system had an enhanced proliferation rate and was similar to the control of the nonprinted cells even after 5 days of cell culture.^147^ In another study, laser‐assisted technique was used for sprinting in situ MSCs onto collagen/hydroxyapatite (nHA) disks to favor bone regeneration in a calvaria defect model in mice. The printed disc demonstrated a larger bone volume after 2 months compared with a cellular collagen/nHA disc.^148^ Keratinocytes were printed on top of 20 layers of fibroblast placed on the top of a matrix Matriderm. Layers of keratinocytes were printed on the matrix, which provided mechanical stability to the overall printed construct. After the complete development of keratinocytes cells, the in vivo study was conducted for 11 days leading to the conversion of cells into a stratified dense tissue when implanted subcutaneously in mice.^146^ The study also demonstrated filling of a 3 mm diameter, 600 μm deep calvarial hole using the composite polymeric biomaterial. Similar polymeric devices is used to fabricate medical components such as a customized, noncellular, bioresorbable tracheal splint, which was used for the patient with localized tracheobronchomalacia.^149^

### 4.4 Other technical approaches

#### 4.4.1 Microvascular printing technique

This technique has attracted a lot of attention for its several applications in drug screening for toxicology studies, fundamental cell biology research, tissue models, and wound generation applications. It consists of a 3‐axis movable robotic platform and an array of multiple electromechanical microvalve print‐heads. Individual gas connection is provided along with the gas regulator to each microvalve print head. It provides a positive pneumatic pressure along with a 0.1 ms valve opening time, which is controlled by the movement of the plunger and the solenoid coil. The magnetic field is induced by the applied voltage, which opens the nozzle by ascending motion of the plunger. The bioink is deposited when the pneumatic pressure overcomes the fluid viscosity and surface tension at the opened orifice. Nozzle diameter, viscosity, and surface tension of the bioink, pneumatic pressure, and valve opening time are vital parameters on which the microvascular process depends ^150^,.^151^ The microvascular technique offers several advantages to the printing process, including the synchronized ejection of biomaterial and cells from various print heads and thin material deposition of about 1–2 μm thickness. Precise cellular positioning is also possible, along with higher viability and high throughput printing of about 1000 droplets per second.^150^ However, the process is disadvantageous during hydrogel printing within a limited range of viscosities. The cell concentration of up to 10^6^ cells per ml is allowed to print as a small nozzle orifice (100–250 μm) can cause clogging during the printing and disrupt the process. The cells do not always disperse and settle over time, which hampers the overall homogeneity of the cells within the bioinks.^151^, ^152^ Homogeneous patterning of cells was facilitated on the thin layers of bioprinted ECM in a controlled area. The 3D printed cell displayed more homogeneous cellular layers with ECM 1–2 μm thickness, whereas the manual seeding cells were of 20–30 μm ECM thickness of discrete multilayered cellular clusters. The 3D printed cells induced higher structural and functional resemblance to the native barrier.^153^ The microvalve technique was used to print organs with improved mimetic nature to the original organs. In one study, this technique was used to print a construct of (6 × 6 × 1.2 mm^3^) by repeated deposition of the collagen precursor. The printed structure was mechanically stable to retain its shape and dimensions compared with the manually seeded complexes, where profound changes in shapes and dimensions were observed during the culture.^152^

#### 4.4.2 Vat polymerization

It is a 3D printing method that involves photopolymerization to cure the liquid ink placed in a vat into a layered volumetric construct. The technique has been commercially available for more than 30 years, used mainly for the fabrication of tissue scaffolds along with the conventional cell‐seeding approach. Stereolithography (SLA) is the primary technique of vat polymerization. It leads to the fast production of volumetric structures with precise internal and external structures in several biomedical applications. This method utilizes a laser beam that helps to sweep around polymerizing single lines of ink in a faster scan mode until each layer has been completed. The SLA system uses a photocurable bioresin to print the construct using top‐down and bottom‐up printing approaches ^154^,.^155^ Another method is used in the Vat polymerization technique, known as digital light‐processing (DLP), which has emerged as an improved method in VP printing. It uses a digital micromirror device (DMD) in DLP, which causes immediate crosslinking of a layer of photocurable resin instead of printing at the single dot in SLA. The DMDs provide the rotation to be in either an ON or OFF position, which helps the photocrosslinking of a bioresin layer. The rotation reduces the build time as the process only depends on the thickness of the layer and the necessary time of exposure.^156^, ^157^ Two‐photon polymerization (2PP) is another technique used in vat polymerization to print the material. The process uses a near‐infrared femtosecond laser (∼740 nm from titanium: sapphire femtosecond laser) to fabricate precise 3D structures with high resolution on the nanoscale. This polymerization process starts with the 3‐order nonlinear absorption within the focal region. The laser beam is tightly focused on the photoresist on a glass coverslip with an oil‐immersion objective lens. Further, it causes fabrication of high‐resolution 3D structures beyond the optical diffraction limit by adjusting the beam within the photoresist.^158^, ^159^ Bioinks like PEG, PCL, PEG‐co‐PDP, PEGDA, PCL/HA, HA/PEEK, and titanium are some of the materials used for the vat polymerization for printing a 3D construct.

The nature of the 3D construct printed is affected by the fabrication strategy of the different bioprinting techniques. The crosslinking process and the crosslinking agents mainly dominate the fabrication of the 3D constructs. Some hydrogels require support while processing, whereas some are mechanically stable to withstand and to complete the process. These fabricating strategies will be discussed in the next section.

## 5 BIOPRINTING FABRICATION STRATEGIES

3D bioprinting is a complex process starting with the material selection and ending with the fabrication of a similar biomimetic structure as proposed. Fabrication deals with the crosslinking strategies used for different bioprinting techniques, as discussed earlier. These fabrication techniques are used to accomplish print reliability and resolution. The rheological property of biomaterial plays an important role in preserving the structural integrity of the printed 3D constructs for cell deposition.

### 5.1 Direct bioprinting

As the name suggests, this fabricating technique prints the desired complex biomimetic structures. The biomaterials are required to have desired rheological and mechanical properties throughout the printing process to attain a similar mechanical and structural complexity. Viscosity, yield stress, and mechanical reliability are a few necessary factors that need to be controlled during and after printing.^128^, ^160^, ^161^ The viscosity of the biomaterial can be enhanced by pre‐exposure of photopolymer to UV light, adjusting temperature and adding enzymes crosslinker.^162^, ^163^ In a few cases, a thickening semi‐crosslinked mixture was used along with the thickening agent to directly print the 3D construct.^164^, ^165^, ^166^ A similar study was performed by synthesizing photocrosslinkable hyaluronan‐gelatin hydrogels for printing a complex feasible tubular construct. The synthesized bioink displayed biocompatibility, supporting cell attachment and proliferation of HepG2 C3A, Int‐407, and NIH 3T3 cells in vitro. These photopolymers when exposed to UV light displayed improvement in the viscoelastic property and thereby similar values of storage modulus (G′) and loss modulus (G″) of the hydrogel.^163^ Zhang et al. directly bioprinted vessels like tubular microfluidic channels. Chitosan and alginate hydrogels were used along with the cartilage progenitor cells and displayed average cell viability of 63% after 12 h of media perfusion postprinting of the complex. The viscosity of the structure was affected by the amount of chitosan used.^167^ Only ∼3% chitosan was feasible to print microfluidic channels with required fabrication parameters as 2% lacked mechanical integrity, whereas 4% was so viscous that it was not coming out properly from the nozzle. The viscosity of the bioink can be altered using thickening agents like hydroxyapatite, nanocellulose, xanthum, and gum, thereby increasing the printability to form a complex 3D structure.^88^, ^164^, ^166^, ^168^ Wouter et al. used gelatin methyacrylamide hydrogels to print tissue‐engineered cartilage constructs. The printed hydrogel was tested with chondrocyte cells; efficient viability and differentiation were seen in cells, and enhancement in mechanical properties was also seen in the hydrogel. Gelatin methacrylate solution has low viscosity at normal body temperature, making it incompatible with biofabrication processes. However, the addition of HA or codeposition with thermoplastic enhanced the viscosity and, thus, favored as a compatible bioink material.^169^ During the fabrication process, the biological hydrogels, composed of polysaccharides and proteins, are difficult to print as the in situ gelation is a critical stage as they need to be provided with support so that they do not collapse or deform under their own weight.^170^ The support bath is used widely to fabricate complex hydrogel‐based overhanging structures. The support bath materials should have a rigid matrix, which yields by a passing nozzle and rapidly recovers itself after the motion of the nozzle. The support bath provides the necessary gelation to the hydrogel before integrating the subsequent layers without clogging the nozzle. Several materials have been used for providing support in the form of a support bath. Pluronic is one of the biocompatible materials playing the dual role as fugitive bioink and support bath due to its thermoreversible sol–gel phase transition property. The mechanical weakness and rapid dissolution are the main concerns with this material as they limit the duration up to which the hydrogel can be supported by the PF baths.^170^, ^171^, ^172^ Wu and his research group modified PF with triblock copolymer to increase its efficiency as a support bath. They synthesized a fluorescent dyed fugitive ink, which comprises an aqueous solution of pluronic F127, a triblock copolymer with a hydrophobic poly(propylene oxide) (PPO) segment and two hydrophilic poly(ethylene oxide) (PEO) segments in a PEO–PPO–PEO fashion was used as a fluid filler. The diacrylate functionalized pluronic F127 solution was used as a physical gel reservoir. The 3D biomimetic microvascular networks with a 3‐generation hierarchical branching topology of 200–600 μm microchannel diameter were constructed using this system. Fumed silica nanoparticles‐based suspension was used as a hydrophobic support bath for the 3D extrusion printing. Hydrophobic silica‐mineral oil suspension was used to deposit hydrophobic inks like PDMS, SU‐8 resin, and epoxy‐based conductive ink. The structural integrity was maintained during printing as well as curing was done up to 90°C. The bath was further able to provide feasibility, versatility, and a much better resolution such as 30 micron for PDMS filaments.^173^, ^174^, ^175^

### 5.2 In‐process crosslinking

Crosslinking is a vital step in 3D bioprinting that influences the printed construct's mechanical, physiochemical, and cellular properties.^88^ Hydrogels with a rapid gelation mechanism are primarily used through the in‐process crosslinking method. This crosslinking is accomplished either by changing the extrusion head for coaxial extrusion of the precursor and crosslinker. This can be obtained by depositing precursor into a crosslinker bath.^176^, ^177^ The crosslinking can be modified by changing the material or by varying the printer's tool path design.^178^, ^179^ Recently, extrusion bioprinters are customized for specific applications, including core‐shell printing, combined extrusion and ES, and UV curing during and postprinting.^113^ Photoinitiators are the critical components of photocurable polymerization systems. Various UV –light photoinitiators are utilized for photocrosslinking. Several limitations like low water solubility, inhibition with oxygen, high‐energy UV light exposure requirement and cell damages have also been reported in this crosslinking process.^180^ Layer‐by‐layer UV curing of bioprinted photocurable GelMA‐based hydrogels was prepared using a rapid extrusion‐based bioprinting technique with an in‐built ultraviolet (UV) curing system. The GelMA improved the bioink printability and shaped fidelity before crosslinking and led to the fabrication of soft tissue constructs with a high aspect ratio (length to diameter) of ≥5. The cell viability of the printed layers was above 80% in all cases.^125^ In another study, water‐soluble photoinitiator lithium phenyl‐2,4,6‐trimethyl‐benzoyl phosphinate (LAP) was emulsified in pentaerythritol triacrylate monomers. Extrusion‐based printing technique was used to print the 3D construct at the optimum concentration of 10 mM and 1.62 M for LAP and triethanolamine, respectively.^180^ Billiet and his coworkers used and optimized VA‐086 as a photoinitiator to fabricate highly viable 3D printed macroporous gelatin methacrylamide constructs.^181^ Alginate and fibrin are among the most common used materials for this type of printing due to their capacity to sustain structural stability and are versatile to be incorporated with any cellular component.^182^ Nishiyama constructed the gel structure using state‐of‐the‐art inkjet technology. Sodium alginate solution was ejected from the inkjet nozzle and mixed with calcium chloride. The nozzle could move in three directions, thereby leading to a new fabrication method for the detailed fixation of 3D gel structures using living biological components.^176^

### 5.3 Postprocess crosslinking

Crosslinking is essential to improve the biomechanical features of the polymers through network bonding. It establishes a connection between two functional groups of a polymer chain through covalent or noncovalent bonding.^183^ However, in a few cases, extrusion technology was used for crosslinking. Still, it cannot be completed before and during the printing as the bioinks may consist of different types of polymer with different crosslinking patterns and mechanisms. In such cases, the printed construct is exposed to crosslinkers after printing. This bioink mixture used in this process is made of two types of materials: primary and secondary. Primary material forms the basic framework and helps to enhance printability and shape accuracy during the complete printing process. At the same time, secondary material undergoes crosslinking after printing to provide structural accuracy.^88^ Seung et al. fabricated alginate scaffolds in a two‐step process; a modified dispensing process and an aerosol spraying method using calcium chloride as a crosslinking agent. A 3D pore‐structured, cell encapsulated alginate scaffold of 20 × 20 × 46 mm^3^ was constructed with varying crosslinker concentrations of the aerosol and dispensing process. The printed construct displayed 84% cell viability to prepsteoblast (MC3T3‐E1) cells.^184^ In another gelatin‐methacrylamide (GelMA)/hyaluronic acid (HA) polymeric system, GelMA alone could not provide the desired structural property and integrity as well as printability. The acid improved the viscosity and therefore enhanced the printed structural 3D construct. The HA also improved the cell viability to 82% compared with the pristine GelMA system toward chondrocyte cells.^169^ Postprocess crosslinking was used to construct heterogeneous aortic valve conduits using alginate/gelatin polymeric mixtures. The constructed aortic valve had viability over 80% for aortic root sinus smooth muscle cells (SMC) and aortic valve leaflet interstitial cells (VIC) (Figure 2B).^76^

### 5.4 Indirect printing

This process involves using a sacrificial agent followed by inducing a crosslinking agent, which gets removed upon final printing of the desired 3D construct. This type of printing commonly uses build/support configuration for printing constructs or models with high structural reliability. Build materials are generally engineered tissue components (cells and/or hydrogels), which support or help base materials to provide mechanical strength to hold the structure with a strong grip 84 tightly. In most cases, the support material may be deposited with the build material but is removed through postprocessing procedures. This process has allowed the use of particular material, which was not favorable earlier due to its characteristics and can be used in indirect printing.^185^ The 3D filament networks printed using carbohydrate glass displayed a rigid behavior. The printed networks were used for the biocompatible sacrificial template in several engineered tissues. These templates were further used to create cylindrical networks. The cylindrical networks were lined with endothelial cells also permeated with blood under pulsatile flow of high pressure. This approach allowed independent control of endothelialization, network geometry, and extravascular tissue.^161^ Alginate and gelatin precursors are mixed with different concentrations of hydroxyapatite to print a 3D complex. It is a two‐step mechanism that combines gelatin's thermosensitive and alginate's chemical crosslinking properties to form the structure.^186^ Materials like gelatin, pluronic F127, and agarose show reversible crosslinking mechanisms, making them suitable for support material.^88^ Alginate was used to construct a scaffold for nerve tissue applications through indirect printing. It involved printing a sacrificial framework from gelatin, impregnating the framework with a low concentration of alginate, and finally, the gelatin framework was removed using an incubation process. The printed lower alginate scaffold has better cell viability and functionality as compared with the higher alginate scaffold.^187^

### 5.5 Hybrid printing

Hybrid printing involves using computer‐aided technology and several other fabrication processes. Multiscale parts have been constructed due to the integration of bioprinting techniques with melt‐plotting and ES apparatus. Kang et al. used a hybrid printing method to fabricate the human‐scale tissue construct with enhanced structural properties. The tissue was constructed by representing clinical imaging data as a computer model of the anatomical defect and then translating the model into a program that can control the motion associated with the printer's nozzle. Additionally, it can help distribute the cells to discrete locations. The microchannel was fabricated inside the tissue constructs, facilitating the diffusion of nutrients to printed cells.^20^ Printing ECM with accurate structural integrity and exact components is critical for several technologies. A large amount of materials has been used for printing the matrix, but the complexity of the natural ECM leads to complications in printing them with intrinsic cellular morphologies and functions. A hybrid method was developed to bioprint the cell‐laden constructs with a novel decellularized ECM (dECM) bioink, which can provide an optimized microenvironment favorable for the 3D growth of tissues.^32^ PCL and PLGA are commonly used as support for hybrid printing. These polymers are generally processed at high temperatures, making it difficult for cells to deposit them directly. In order to overcome this factor, cells are deposited onto them using hydrogel as a carrier.^88^ PCL/alginate hybrid scaffolds were fabricated using the aerosol crosslinking process for even cell distribution (Table 3). Scaffolds can provide repeated and appropriate pore structure but low biological activity, low cell‐seeding efficiency, and nonuniform cell density. PCL scaffold coated with alginate provides enhanced cell viability, cell seeding efficiency, and cell distribution for prosteoblast cells (MC3T3‐E1).^188^, ^189^

**TABLE 3:** Bioprinting techniques and their crosslinking strategy

Bioprinting technique	Crosslinking method/agent	Bioink	Target tissue	Reference
Inkjet	Ionic‐CaCl_2_	Alginate	Bone tissue	^190^
	Ionic‐CaCl_2_	Alginate	Not specified	^191^
	Thermal Pre‐gelation + ionic‐CACL_2_	Alginate/gel	Not specified	^192^
	Ionic‐SrCl_2_	Alginate/Guar Gum	Human cartilage	^166^
	Electrostatic interaction/hydrophobic	Peptide/keratin	Not specified	^193^
	Hydrogen binding + π–π stacking	Supramolecular‐PCL	Not specified	^194^
	UV crosslinking/(Irgacure 2959)	PEGDA	Cartilage tissue	^195^
Extrusion	Ionic‐CaCl_2_	Alginate	Cardiac tissue	^196^
	Thermal Pre‐gelation + ionic‐CaCl_2_	Alginate/gel	Lung	^113^
	Ionic‐CaCl_2_	Alginate/guar gum	Not specified	^197^
	Ionic‐CaCl_2_	Alginate/PCL	Human cartilage	^198^
	Electrostatic interaction	PEG/Clay	Not specified	^199^
	Electrostatic interaction/ UV crosslinking	GelMA/κCA /Clay	Not specified	^200^
	Electrostatic interaction	Gel/Ch	Skin	^27^
	Hydrogen bonding	Gly/Clay	Bone regeneration	^201^
	DNA hybridization	Polypeptide/DNA	Not specified	^202^
	UV crosslinking/(Irgacure 2959)	GelMA/Silicate	Bone tissues	^203^
	UV crosslinking/(Irgacure 2959)	GelMA/CNT	Myocardial tissue	^204^
	Ionic‐CaSO_4_	Ch‐HAp	Bone tissue	^205^
	Electrostatic interaction	Alg/MC/Clay	Bone tissue	^206^
Laser‐assisted	Ionic‐CaCl_2_	Alginate	Skin tissue	^207^
	Thermal pre‐gelation + ionic‐CaCl_2_	Alginate/gel	Not specified	^208^
	Electrostatic interaction	HA	Corneal structures	^209^
	Visible‐light crosslinking/rose bengal	Tyramine‐HA	Not specified	^210^
Stereolithography	Hydrogen binding + UV crosslinking	PCL‐Pyrimidinone	Not specified	^211^
	UV crosslinking/(LAP)	GelMa	Vascular tissue	^212^
	UV crosslinking(Irgacure 2959)	GelMA/PEGDA	Cartilage tissue	^213^
	Photo/Lap	GelMA/PEGDA	Spinal cord	^214^
	UV crosslinking/(Irgacure 2959)	GelMA/M‐HA	Adipose tissue	^215^
	Visible‐light crosslinking/(VA‐086)	PEGDA	Not specified	^192^

In the 3D printing design of organs or tissue, 3D models allow the researchers to create a functional prototype of the model faster than any other prototyping process. This also enhances the research efficiency as less time is consumed by these prototype models used for different works. Several factors affect the design factors and capabilities of bioprinting, which can be listed and explained in the next section.

## 6 DESIGN FACTORS AND CAPABILITIES OF BIOPRINTING

Bioprinting technology uses computer‐aided technology to create engineered tissues. The software‐based technology has allowed greater precision and control while printing as it has allowed variation in terms of cell‐material shape and deposition along with the modification in single or multiple depositions of the cells on a single substrate. This digital technology has allowed the introduction of digital analysis to understand the printing process at a super refined and cellular level. This understanding has helped fabricate even a complex structure like skin with different cell densities and cell types printed on the same substrate.^88^

### 6.1 Shape and resolution

3D printing is one of the advanced, evolving technology known for creating or printing novel or biomimetic 3D complex constructs. It helps to produce a replica of a structure similar to the original one with great resolution and precision. It helps to create unique 3D constructs with the help of biomaterials and cells, thereby replacing the orthodox approach of using metals and certain plastics to create 3D models. The shape of the printed 3D constructs must be anatomically precise in order to replace the required organ, cellular or tissue model.^88^ Several factors like printing technology, biomaterial, and fabrication strategy affect the shape of the 3D construct to be printed. Printer resolution, accuracy, and platform size for printing also affect the shape and features of the desired printed 3D constructs (Figure 2C).^76^, ^181^ 3D bioprinting needs a computer and computer‐aided design to fabricate the assigned 3D design perfectly. SL plays a vital part in this 3D printing for describing the surface geometry of a 3D model or object by creating a series of linked triangles. Triangles are linked in a serial order for better resolution. It plays a significant part in accurately printing the desired 3D constructs.^216^ Mannor et al. constructed a 3D printed bionic ears with its electrical components, which increased the printed ear's efficiency and broadened human hearing capability.^217^ Duan et al. attempted to create a 3D printed heart valve using an extrusion‐based printing method, and the reverse engineering approach has been exploited for this method. A porcine aortic valve was harvested, and the data were imported into an open‐source bioprinter and anatomically correct the aortic valve using this approach.^76^ In another work, heart valves were drawn into solid work and were printed afterward. 3D bioprinting helps print an anatomically correct virtual shape with reasonable accuracy and precision. Resolution is one of the properties while considering good image. In 3D printing, resolution refers to the smallest movement of the nozzle, which can deposit a material on the substrate with better precision. The 3D printers have two types of resolution XY resolution and Y resolution. The resolution of the horizontal movements of the printer can be controlled by XY resolution, and the Y resolution governs the resolution of the vertical movements associated with the printer.^218^ The smallest material unit constructed affects the resolution and accuracy of the printed 3D constructs. The layer function and thickness affect the resolution in the Z direction. Printing further layers in this direction will affect the overall structure. Sometimes the printing path height, path space, and nozzle diameters also cause a change in the resolution of the printed 3D constructs.^219^, ^220^

### 6.2 Material heterogeneity

The bioprinting phenomenon involves the basic approach of using biomaterials, cells and growth factors to print a desired 3D complex construct of specific cellular, tissue or organ model for the treatment of specific infections or diseases. However, these components can vary based on the necessity and specificity of the biomedical applications, which can lead to heterogeneity in the cells, materials and growth factors required to print a desired 3D construct.

#### 6.2.1 Cells

3D printed materials consist of two vital components, biomaterial, which will form the skeletal framework of the printed construct and live cells, which needs to be incorporated within the framework to create a replica of the proposed structure, organ or model. Single cells, cell aggregates and multiple cell types can be varied while utilizing the cells. Cells that are young and immature take on individual characteristics and become mature to form specialized cellular forms and function.^221^ The specialized human cells that have the ability to differentiate into various cell types are known as stem cells. They have been used in regenerating many organs and tissues in the human body for their unique properties. Pluripotent and multipotent stem cells have been widely used in 3D bioprinting. Pluripotent stem cells are the specialized cells having the ability to undergo self‐renewal. All the cells of the tissue of the body can be generated by these stem cells. In contrast, multipotent stem cells have the capacity to self‐renew. This renewal is done by dividing and can develop into multiple specialized cell types present in a specific tissue or organ.^222^ Schuurman et al. worked on printing PCL fibers with alginate constructs using extrusion‐based printing, and the mechanical property was varied by changing the fiber spacing, orientation and thickness. Cell viability was calculated using C20A4 cells, and cell viability became high immediately after printing and was close to 75% even after three days of in vitro culture.^189^ Zhu et al. constructed gelatin methacrylamide hydrogel with graphene nanoplatelets using SL printing. This model was used to provide a biocompatible microenvironment for survival and growth, leading to the successful construction of neural stem cells. Well differentiation, architecture and homogeneous cell distribution were observed in the printed cells.^223^ Seol et al. fabricated a 3D printed biomask for facial skin wounds and reconstruction. The layers of skin were constructed using an extrusion‐based bioprinting technique. A porous PU layer, a keratinocyte‐laden hydrogel layer and a fibroblast‐laden hydrogel layer were used to create different layers of skin. Human epidermal keratinocytes and human fibroblasts were used to study the biological activities. In the animal study, the subcutaneous implants reduced the wound area by <40% after 14 days and also the epidermis and dermis layer were regenerated using this technique.^224^

#### 6.2.2 Biomaterials

3D bioprinting is a blooming and flourishing technology in the field of medical application to create similar structures of different cellular, tissue, and organ models. It is used to create scaffolds, orthoses, and prosthetic devices for different biomedical applications. Several natural and synthetic materials are used for printing 3D constructs. Printable materials should have adequate viscosity, form 3D structure in a definite time, and be mechanically reinforced, have tunable mechanical properties, be biocompatible, have adequate degradation kinetics, and be nontoxic degradation byproducts, biomimetics and property to release therapeutic agents in a controlled manner. The biomaterials should be affordable, commercially available as well as can easily be manufactured and processed.^52^ Natural polymers like alginate, gelatin, chitosan, collagen, silk, HA, fibrinogen, and agar have been used alone or reinforced with other polymers/fillers for creating 3D complex models. Synthetic polymers like ABS, PLA, PGA, PLGA, PU, polyamides, and several other polymeric hydrogels can be used as biomaterials for creating biomimetic constructs ^51^, ^84^,. Yu Hsieh synthesized PU nanoparticles and used them with neural stem cells to print a 3D structure using extrusion‐based bioprinting technology. It did not use heat, toxic organic solvents, photo initiators, or ultra‐visible light for crosslinking. This printed structure was successfully implanted in adult zebrafish and helped repair the traumatic brain injuries, and function was restored.^225^ Fibrin is a tough protein used in several biomedical applications due to its extraordinary structural and mechanical properties. It was used with collagen to bioprint amniotic fluid‐derived stem cells. The structure was found to help increase the rate of healing of large skin wounds as they quickly closed full‐thickness burns and enabled revascularization of tissue.^226^ In another case, fibrinogen was used for tissue engineering application to print engineered thick tissues successfully. Human microvascular endothelial cells (HMEVs) were incorporated with fibrin to act as a base for microvascular construction. Confluent lining were seen by the alignment and proliferation of cells aligned inside the channels.^227^ In another study laser‐assisted bioprinting technique was used to form a skin‐like structure in the dorsal skin fold chamber in mice. Skin substitutes were created by positioning fibroblasts and keratinocytes on top of a stabilizing collagen matrix. The transplants were positioned in full‐thickness skin wounds, and full connection to the neighboring tissue was observed after 11 days.^146^

#### 6.2.3 Growth factor

Growth factors are the group of proteins that are responsible for the regulation of proliferation and differentiation of cells to encourage tissue regeneration. Growth factors can control degradability and can protect enveloped molecules from degradation. Hydrogels are often used to regulate the release of growth factors inside the printed structure. Due to high water content, hydrogels support the encapsulation of hydrophilic molecule‐like growth factors without aggregating and denatured.^228^ The growth factors are printed, and their successful implementation requires the preservation of bioactivity of these growth factors and their controlled release after printing.^229^ Freeman used alginate based MSC‐based printed system for the controlled growth factor delivery. Vascular endothelial growth factor was used for the alginate‐based printed construct. After printing the 3D complex, only 45–75% of the growth factor was initially loaded onto the alginate present. The molecular weight of alginate affects the retention capacity of the growth factor. Lowering alginate molecular weight significantly reduces the growth factor retention capacity inside the construct after printing (*p* < 0.05).^9^ Poldervaart studied the controlled release of vascular endothelial growth factor from gelatin microparticles. The growth factors may cause a significant challenge in delivery, but in this study, the printed complex increase its life span. Thus, growth factor was released for three weeks during in vitro studies, and biological activity was also observed upon using human endothelial progenitor cells (EPCs) in migration assays. The prolonged‐release enhanced the vascularization in the printed scaffold.^230^ Growth factor has affected the vascularization of cells in the printed 3D constructs. Gelatin and sodium alginate have similar structural and chemical similarities, which is the main reason behind their use to generate efficient in vitro cell‐laden 3D ECM mimics. Mouse plantar dermis and EGF were combined into the printed mimics to form a specific area for epidermal progenitor cells acquired from mice. The printed 3D structure maintained cell viability and facilitated cell spreading and matrix formation.^231^ Castro et al. used poly(d, l‐lactic‐co‐glycolic acid) nanoparticles in PEG diacrylate with nanohydroxyapatite for 3D printing of biomimetic osteochondral scaffolds. Chondrogenic transforming growth factor β1 was incorporated with core‐shell PLGA nanospheres for sustained delivery. The growth factors induced a synergistic effect, which caused an increase in cell adhesion and directed stem cell differentiation (Figure 4B).^232^ In another study, Bone morphogenetic protein‐7 was used as a growth factor to study the integrated cementum formation on the root surfaces of human teeth using growth factor releasing scaffolds. These printed scaffolds were used with periodontal ligament stem/progenitor cells (PDLSCs). After 6 weeks of study, the resurfacing of dentin was observed in growth factor delivering groups compared with control.^233^ 3D printing has been helpful in various sectors such as manufacturing, industrial design, jewelry, architecture, engineering, medicine, and many other fields.^234^ According to Formlabs, over 90% of the top medical device companies used 3D printing to produce an accurate medical device prototype. Advances in 3D printing technology have made a tremendous contribution to healthcare. New tools, designs, and therapeutic methods have helped patients to bring new degrees of comfort and specificity to the treatment.^235^ Surgical applications, disease modeling, medical devices, implants, prostheses, and tissue engineering are some areas where 3D printing is used in biomedical applications.^236^ In the next section, we discuss the application of 3D printing in cancer and several tissue engineering applications.

## 7 APPLICATIONS OF BIOPRINTING IN THE BIOMEDICAL FIELDS

**FIGURE 3:**
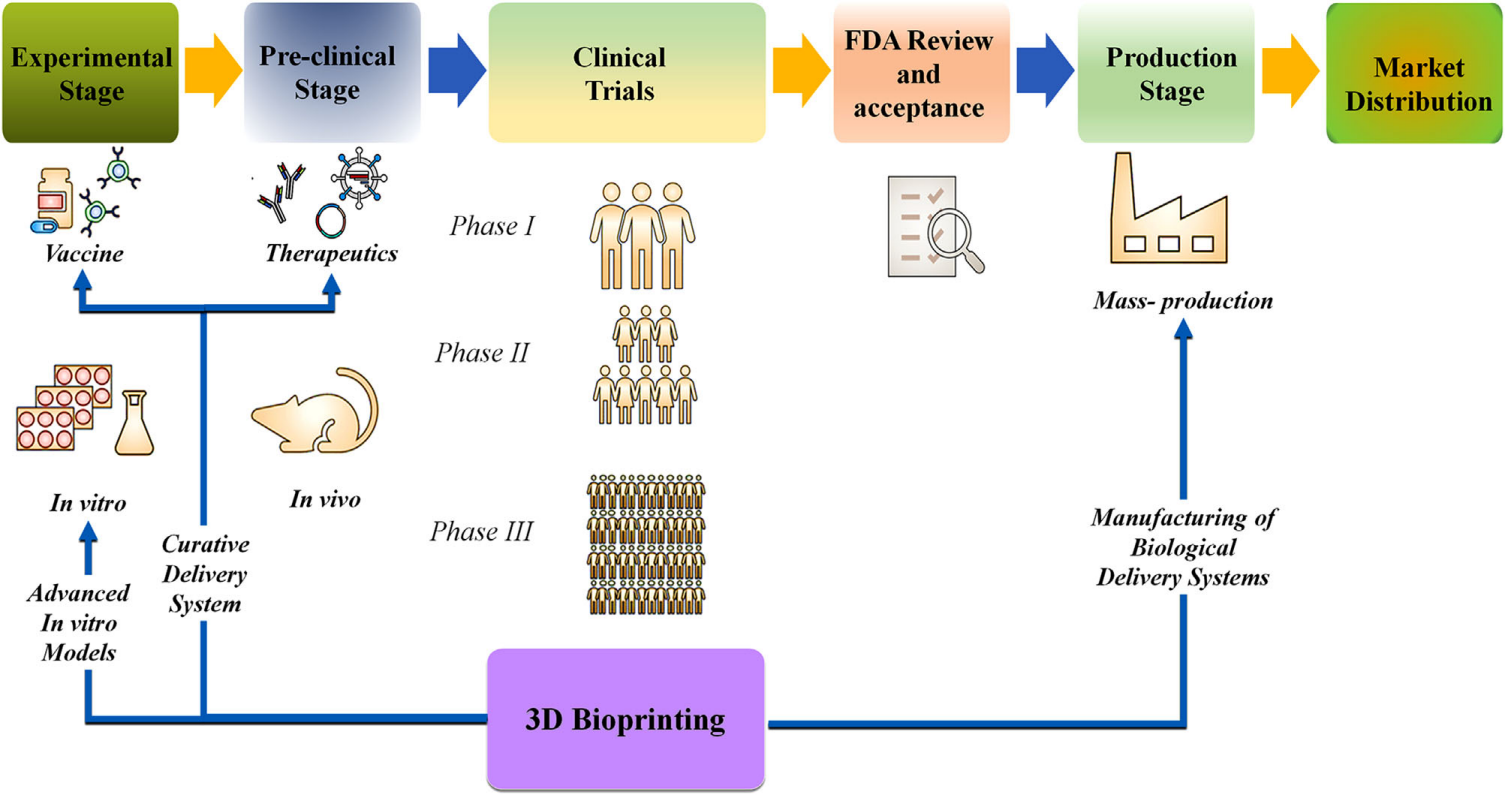
Discovery pipeline for conventional and 3d bioprinting technologies mentioning the processes involved reproduced with permission from Ref. 237, copyright 2021 The Author(s).

Conventional models like in vitro assays and in vivo animal model studies are not fully enumerated the critical characteristics of human physiology. Recent advancements in 3D bioprinting technologies allowed witnessing better inventions in biomedical fields. Application of biocompatible matters and cell supporting components holds promising results for reconstituting cancer microenvironment, tissue engineering, in vitro drug screening and wound healing, skin and vascular regeneration, and so on. 3D printing enhances the deep knowledge of various disease mechanisms and phenotypic viability. 3D printing has shortened the process of any drug discovery and application while going through several phases and clinical trials. The construction and refinement in the models of various diseases can decrease the trial and error involved with the conventional approach (Figure 3).^237^ It has served as a beneficial short approach, which has helped in application in several biomedical fields by constructing desired organ or tissue models to study the medical problems closely on a cellular level.

### 7.1 Cancer therapy

Cancer is one of the leading life‐threatening diseases in the world, accounting for approximately 10 million deaths in 2020 ^238^,.^239^ The most common cancer types spreading worldwide are lung, breast, prostate, and bowel cancer. There will be 27.4 million new cases annually by 2040 if the issue is not taken seriously. To better understand the genesis and progression, we need extra complex and physiologically more relevant models, which closely resemble the in vivo tumor microenvironment. The cancer cells are healthy cells that become oncogenic in the presence of several factors like genetic mutations. These healthy cells upon conversion show capabilities and properties specific to cancer including invasive behavior, escaping growth, avoiding cell death, suppression, and triggering abnormal angiogenesis ^240^,.^241^ Bioprinting has the ability to provide highly controlled cancer tissue models, which can prominently accelerate cancer research. The tumor cells control their propagation and movement by creating proangiogenic factors (e.g., vascular endothelial growth factor, VEGF), and promote their interactions with the stromal cells and the surrounding inflammatory cells including macrophages, neutrophils, and mast cells.^242^

**FIGURE 4:**
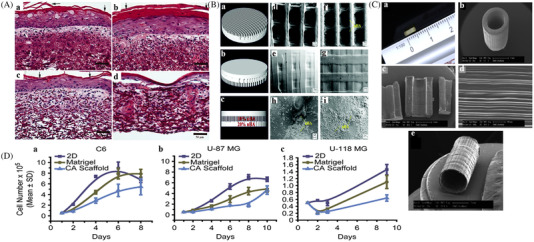
Applications of 3D bioprinting: (A) Stained cross‐section of skin substitutes made (a) 10, (b) 12, (c) 14, and (d) 16% (w/v) gelatin scaffolds after 14 days in culture; reproduced with permission from Ref. 274, copyright 2008 John Wiley & Sons, Inc.. (B) (a)–(c) 3D CAD Model. SEM images of (d) and (e) control scaffolds without nHA (bottom and top images); and (f)–(i) osteochondral scaffolds with graded nHA (f) is the bottom, (g) is the top; h is 10% nHA layer, and (i) is 20% nHA layer reprinted with permission from reproduced with permission from Ref. 232, copyright 2015 Royal Society of Chemistry. (C) (a) Optical, (b) SEM images of PEG nerve guidance tubes of 5 mm (length) × 1.5 mm (diameter) and 250 μm (wall thickness), (c) and (d) SEM images of trenches set of 5 mm long, (c) 13×, (d) 200× magnification, (e) 50 micron thick wall experimentally constructed PEG nerve guide; reproduced with permission from Ref. 288, copyright 2015 ‐Elsevier. (D) CA Scaffold ability to offer in vitro growth environment for tumor cells. (a) The proliferation of C6 Cells, (b) U‐87 MG cells, and (c) U‐118 MG‐Glioma cells were cultured on 2D 24‐well culture plates, Matrigel matrix, and CA scaffolds. The cells were cultured for up to 8–10 days and reproduced with permission from Ref. 248, copyright 2010 ‐Elsevier.

The heterogeneity and complication of the tumor microenvironment can be copied by simultaneously printing bioinks with different cell types, ECM, and biomolecules ^243^, ^244^ ^.^ Various tumor microenvironment models like blood vessels, ECM, cell culture, organoid culture and cancer‐on‐a‐chip are used in 3D bioprinting in cancer therapy (Table 4). By fabricating heterogeneous constructs (containing ECM, different cell types and biomolecules) via bioprinting, one can achieve a great degree of dimensional control to imitate physiologically appropriate cell‐cell interactions. By reformulating the bioink, one can study various cancer patterns by printing diverse cancer cell types and neighboring cellular matrix. Zhao et al. ^245^ reported a study in which they applied a 3D bioprinting technology to build in vitro cervical tumor models with Hela cells and gelatin‐alginate‐fibrinogen hydrogels. All the parameters like Cell proliferation, multiphoton polymerization (MMP) protein expression and chemoresistance were matched with the conventional 2D planar culture models (Table 4). This study has shown the consequence of the printing parameters on cell viability and achieved cell viability of over 90%. HeLa cells displays a greater production rate in the printed 3D environment and tended to form cellular spheroids, but in 2D culture, they formed monolayer cell sheets. In 3D printed models, Hela cells performed higher MMP protein expression and chemoresistance than in 2D culture. The printed 3D models have extra simulated tumor characteristics in comparison with the 2D planar cell culture models. Those 3D biological characteristics may benefit while studying biology of 3D tumor.^246^ Mehdi Shakibaei and coworkers (Figure 4D) ^247^ demonstrated that alginate provided an exceptional tumor microenvironment and highlighted that curcumin intensifies and chemo sensitizer HCT116R cells to 5‐Floro Uracil ‐based chemotherapy, which may be efficient for the treatment of colorectal cancer and to overcome drug resistance. Alginate helps CRC cells encapsulate in it, and then it proliferates in 3D‐colon spheres as a Vivo‐like phenotype and is seized from alginate.

**TABLE 4:** List various tumor microenvironment models for 3D bioprinting, mentioning bioink and technology

Types of tumor model	Tumor microenvironment and cell types	Bioink	Technology	Result	References
Blood vessels	Human umbilical vein endothelial Cells,10T1/2 cells (multipotential cell line)	Poly (ethylene glycol), glass, alginate, fibrin, carbohydrate, and matrigel.	Extrusion	The engineered tissues used a 3D stiff filament network of carbohydrate glass as a sacrificial template. Further, the tissue was incorporated with live cells. Glass was utilized to build cylinder‐shaped networks that endothelial cells could line.	^251^
	C3H/10T1/2, clone 8 cells and GFP‐expressing human neonatal dermal fibroblast cells, Human umbilical vein endothelial cells and RFP‐Human umbilical vein endothelial cells.	Poly (ethylene glycol) diacrylate and methacrylated gelatin	Extrusion	A diverse, vascularized and accessible tissue model was constructed.	^32^
	Mouse epidermal fibroblasts (MDFB)	Gelatin‐based alginate, carbon nanotube	Extrusion	The vessel was constructed using gelatin/sodium alginate/carbon nanotube hybrid hydrogel horizontally by communal management of the nozzle and cylinder.	^252^
	Mouse macrophages cell/ mouse glioblastoma cells.	Poly (ethylene glycol) diacrylate and methacrylated gelatin−gelatin bioink	Extrusion	A 3D in vitro sacrificial bioprinting model of glioblastoma was constructed. The model contained vasculature features for cellular study in the 3D microenvironment.	^253^,^254^
ECM	Human umbilical vein endothelial cells, human dermal fibroblasts	Agarose and type I collagen, fibrinogen	Microvalve based	The agarose, type I collagen and fibrinogen mixtures were used for 3D printing. The capillary‐like network formation was also constructed with different cells.	^255^
	Chondrocyte and ECM	Collagen type II	Extrusion	The hydrogel construct of collagen type II and biomimetic chondrocyte density gradient displayed biological effects on the cell circulation and the total cell density.	^256^
	Human adipose‐derived stem cells (ADSC), human endothelial colony‐forming cells (ECFCs)	Fibrinogen	Laser printing	Using laser printing, a fibrin layer‐by‐layer approach was used to create 3D arrays ASCs and ECFCs. This structure demonstrated a vascular‐like network, also cell contact with the ECM molecules.	^257^
	Mouse embryo cell (10T1/2) and HeLa (cervical cancer cell)/human mammary epithelial cell (HMLE)	Poly(ethyleneglycol) di‐acrylate (PEGDA)	DMD‐PP(digital micromirror de‐vice‐based projection printing)	The system was used to print designated required complex structures within a short time.	^258^, ^259^
Cell culture	IMR‐90 fibroblast cells and MDA‐MB‐231 cancer cells	Alginate and gelatin	Extrusion	The complex printed models were used for long culture (>30 days). The printed models had predictable size, frequency, and heterogeneity of multicellular tumor spheroids	^260^
	Pancreatic cancer and stellate cells, endothelial cells	Alginate and gelatin	Extrusion	Pancreatic cells were printed with the microenvironment. The printing occurred only at a very high concentration.	^261^, ^262^
	Luminal(MCF‐7), basal‐like(HCC1143), HER2 amplified (SKBR3), and claudin‐low (MDA‐MB‐231)	Alginate and gelatin	Extrusion	The complex construct was used to print multiple cell types. These cells included cells derived from the patient onto scaffold‐free in vitro tumor tissues.	^263^
Organoid culture	Immortalized nontumorigenic human breast epithelial cell lines MCF12A and MCF10A	Rat tail collagen I	Extrusion	This method was used to print models with excellent repeatability and reliablility. Good coordination was also seen for large luminal models in cluster positioning.	^264^
	Immortalized non tumorigenic human breast epithelial cell line (MCF‐12A), breast carcinoma cell lines (MCF‐7) and MDA‐MB‐468.	Rat tail collagen	Extrusion	Increased tumoroid formation was seen with particular 3D arraying. Coprinting of cancer cells and mammary epithelial cells resulted in the generation of chimeric organoids.	^245^
Cancer cells‐on‐a‐chip	Hepatocellular carcinoma cell line (HepG2) and human malignant glioblastoma multiform	Alginate	Inkjet	Highly accurate patterning with several cell types was seen in this method. The structure had the capability for microfluidic integration.	^265^
	Hepatocellular carcinoma cell line (HepG2).	polydimethylsiloxane, alginate hydrogel	Extrusion	The direct cell writing fabrication phenomenon was used to produce a 3D cell encapsulated alginate‐based model. This model was used to study various pharmacokinetic parameters.	^266^
	Human umbilical vein endothelial cells, lung cancer cells A549	Poly (ethylene glycol) diacrylate and methacrylated gelatinpoly (lactic‐*co*‐glycolic) acid, AuNR	Extrusion	The printing technique was used to fabricate tumor constructs by accurately settling live cells, biomaterials and programmed releasing capsules.	^267^

During the growth of cells in alginate, isolation of three stages of cells, namely proliferating alginate, invasive and adherent cells, are observed. Tumor‐assisting parameters (CXCR4, MMP‐9, NF‐κB) were considerably expanded in the increasing and invasive as compared with the adherent cells and the parental HCT116, HCT116R (human colon cancer cells) over expressed, indicating a rise in malignancy behavior. In alginate, curcumin intensified 5‐FU‐induced reduced capacity for proliferation and invasion and improved sensitivity to 5‐FU of HCT116R in comparison with the HCT116 cells. Treatment with 5 μM curcumin considerably reduced 5‐FU concentrations in HCT116 and HCT116R cells to (0.8 and 0.1 nM, respectively) instead of providing 8nM concentration in both the cases by downregulation of NF‐κB activation and NF‐κB regulated gene products. Kievit et al. ^248^ used the Human U‐87 MG, U‐118 MG glioma cells, and rat C6 glioma cells for the study. U‐87 MG and U‐118 MG cells exhibit notably higher malignancy when cultured in CA (Chitosan‐alginate) scaffolds, while the behavior of C6 cells showed minimal differences because of their malignant and invasive nature (Figure 4A). CA scaffolds came up with a better prototypical 3D microenvironment for glioma cells, which was symbolic for in vivo tumors and thus can offer an effective tool for studying anticancer therapeutics. These unique CA scaffold tools may provide an alternate pathway to the sluggish and expensive animal studies for an extensive selection of experimental designs.

Xiaolan Fang et al. ^249^ established a novel double‐layered alginate hydrogel microspheres in a 3D coculture model; in separate compartments of the microspheres, prostate cancer epithelial and stromal cells were incorporated. Over 30 days, these cells remained confined and viable within their respective spheres. To prove the principle of paracrine function of the model, the shedded component of E‐cadherin (sE‐cad) (a significant membrane‐bound cell adhesive molecule that is highly dysregulated in cancers, including prostate cancer) had been measured in the conditioned media. In addition, sE‐cad can reliably be quantified by epithelial–stromal interaction. Further, this study provides a novel 3D in vitro coculture model helpful in studying cell‐to‐cell paracrine interaction. Patel et al. worked on AlgiMatrix ^250^ scaffolds forming multicellular spheroids in size range of 100–300 mm. Several anticancer drugs were separated by incubating them for 24 h at 7, 9, and 11 days in 3D cultures. The Cytotoxicity of the scaffolds were examined with the help of the Alamar Blue assay. Spheroid number and size distribution showed the success of anticancer treatments. The results of conventional 2D monolayer cultures suggested that IC_50_ values for anticancer drugs were considerably greater in AlgiMatrix^TM^ systems for the 3D model as compared with 2D culture. The cleaved caspase‐3 expression in H460 spheroid cultures was significant (2.09 and 2.47 folds for 5‐fluorouraciland and camptothecinin, respectively) compared with 2D culture. The cytotoxicity, spheroid size distribution, immune‐histochemistry, RT‐PCR and nanoparticle penetration data suggested that in vitro tumor models provide higher resistance to anticancer drugs and indicating 3D culture is an enhanced model for the cytotoxic evaluation of anticancer drugs in vitro. These findings are breathtaking to progress toward a high output in vitro tumor models to study several effects of various anticancer formulations.

### 7.2 Tissue engineering application

The human body comprises four different tissues, namely connective, nervous, muscular, and epithelial, which unite to perform a specific function. The function of each tissue depends on its architecture, biochemical composition, and mechanical properties. The primary purpose of any biomedical research is to develop more effective modes of diagnosis to cure human diseases and to understand molecular basics in a better way. Nervous disorders have reached almost one million worldwide, which includes traumatic brain disease, neurodegenerative disease, spinal cord injury, autism, Parkinson's disease, Alzheimer, and so on.^268^ The need for tissue regeneration and organ replacement has spurred quickly in recent years, although the number of donors is still insufficient. The classical animal model has been used in many cases and shows signification results. However, a recent study shows that the disease manifestations and phenotype is not similar in mice and human.^269^, ^270^ The inability to perfectly mimic the human underlying molecular mechanisms is evident and gradually increasing in many animals, which is the reason behind the failure of drugs to achieve therapeutic safety and efficacy in clinical trials. Other drawbacks include costly animal experiments, time consuming, and cannot fully monitor the phenomena at the cellular level.

Meanwhile, pharmaceutical companies are emphasizing the investigations on cultured human cell lines.^271^ 3D bioprinting offers a promising solution to the unmet tissue and organ regeneration demands and pharmaceutical research demands. Tissue engineering, a branch of biomedical engineering that targets to develop tissues and biological substitutes outside the body and then implant them into the site of the damaged part to recover, cultivate, and nurture the injured organs or tissues. Different types of bioprinting technologies have several possibilities in tissue engineering applications.

#### 7.2.1 Skin tissue

The skin separates internal organs from the external surroundings; the most extensive body tissue protects the body from UV light absorption, hydration, heat regulation, and so on. Human skin has a multilayered structure with epidermis, dermis, and hypodermis. Synthetic and substitute human skin is fabricated in the research lab. The two main reasons for false skin requirements are wound healing of burned victims and testing of cosmetics and drugs. The classification of wounds depends on the parameters like depth, area, infection type, and so on. In the case of skin regeneration using 3D bioprinting, the methods used are top‐down and bottom‐up approaches.^272^ In most cases, the top‐down approach starts from a bulk material, the scaffold. Some parameters that control the scaffold properties are biodegradability, biocompatibility, pore size and shape, mechanical strength, and surface topography. Biologically active materials are incorporated into this scaffold. Other techniques like layer‐by‐layer biofabrication, fused deposition modeling, laser sintering and three‐dimensional printing are also included in the top‐down approach. In the bottom‐up approach, microscale or nanoscale molecular assembly is deposited into a macroscopic system. Smaller assemblies can be tissue spheroids, polymer microbeads, or cell aggregates. The selection of materials for 3D bioprinting is a vital step, and biopolymers should be biocompatible and biodegradable. Composite biopolymers have convenient features for 3D bioprinting.^273^ Powell et al. (Figure 4A) ^274^ proposed a foraminous fibrous gelatin scaffold developed through ES. The porosity, diameter of the fiber, and interfiber distance of the scaffolds may vary for different grades of scaffolds. Interfiber space larger than 10 μm shows excessive cell penetration at the initial stage, while a distance between 5 and 10 μm shows more significant cell organization, excellent barrier formation, and good cell viability. Augustine et al. ^275^ invented poly(ε‐caprolactone) based membrane using the ES technique. The membrane was implanted in the wounds of guinea pigs, and complete wound healing takes 35 days after the implantation of the PCL membrane. Powell et al. ^276^ formulated collagen scaffold using ES and freeze‐dried (FD) method. In vitro studies of scaffolds prepared from both methods showed similar cellular organization and cell proliferation responses. Both the scaffolds were implanted in the wound of athymic mice. A high grafting rate was observed in both cases, 87.5% in the case of the FD scaffold, whereas nearly 100% grafting was observed for the ES collagen scaffold. Silk fiber is a natural fiber with biodegradability, good mechanical strength, and attractive resilience, which is spun out from spiders, flies, moths, and scorpions.^277^ Jeong et al. ^278^ prepared silk fibroin nanofiber by ES method. The plasma treatment of the electrospun nanofiber was observed in the methane and oxygen environment. Plasma treatment in an oxygen environment indicates enhanced nanofiber hydrophilicity, whereas hydrophilicity diminishes in methane plasma treatment. Ng et al. discussed 3D bioprinting of pigmented skin, the fabricated skin acquired from distinct types of skin cells. As skin donors, they manifest nearly the same constitutive pigmentation. From the stratified epidermal layers' point of view, pigmented skin has a better analogy to native skin than the manually cast ones.^279^ 3D extracellular bioprinting niche gives rise to epidermal stem cell (ESC) discrimination into sweat gland (SG) cells. For SG generation, ESCs were appraised as seed cells because SGs are obtained from embryonic ESCs.^280^ The stem cells mainly used for SGs regeneration are bone marrow stromal cells (BMSCs), mammary progenitor cells (MPCs), and MSCs.^281^ Liu et al. manifested 3D bioprinted gelatin‐based hydrogel matrices with precise pore structures. They inspected the differentiation of hydrogel encapsulated epithelial progenitors (EPs) and their responses to probable self‐organization of SG formation in 3D constructs.^282^

#### 7.2.2 Vascular tissue

The sacrificial bioprinting technique is the most commonly used technology for vascularized tissue engineering. This technology is a four‐step process; deposition of bioink, casting, mechanical extraction, and endothelialization. Lee et al. fabricated a single vascular channel within a 3 mm deep collagen scaffold. Under physiological conditions, the tissue viability of the track shows a distance of 5 mm and a density of 5 million cells /ml ^283^. Zhu and coworkers developed prevascularized tissues using a unique bioprinting approach called microscale continuous optical bioprinting. This study used GM‐HA and gelatin methacrylamide (GelMA) hydrogels, and C3H/10T1/2, human umbilical vein endothelial cells (HUVECs) cells were included in the gels. This combined system was then bioprinted into a vascularized structure, and wealthy endothelial network formation and cell viability were observed in this bioprinting method.^212^ Another study highlighted an interconnected and three‐dimensional vessel network that links to the host vascular after one day of grafting. This study developed a three‐dimensional network through the coculture of fibroblast and endothelial cells (ECs) in a fibrin gel medium. Blood endothelial progenitor cell‐derived ECs (EPC‐ECs) vessel was formed in the subsistence of fibroblast. Increasing fibroblast density from 0.2 million cells/ml to 2 million cells/ml showed increased vessel network formation in vitro and superiority in vivo anastomosis.^284^ Gang et al. reported that vascular channels engrafted to the host vessels by using the wrapping and tapping method. In this process, host vessels were wrapped up by the implanted ECs first, and then the other functions like pericyte reorganization and underlying host displacement occur.^285^ Recent advancements of engineered vessel networks comprise high aspect ratio tubular structures production.^137^ The vascular tissue engineering approach is nowadays more effective in the field of clinical studies as compared with prosthetic grafts.^286^ Meyer et al. synthesized α,ω‐polytetrahydrofuranether‐diacrylate (PTHF‐DA) resin, which has a light activation property. By applying MMP and SL techniques smallest vascular supplying system of 10–100 μm was made.^287^

#### 7.2.3 Neuronal tissue

In the field of neuronal tissue regeneration, 3D bioprinting has a tremendous prospect. Patman and coworkers applied the micro‐SLA technique to fabricate a photocurable resin of PEG (Figure 4C).^288^ The device has assertable handling properties and displayed autograft control properties in thy‐1‐YFP‐H mouse after 21 days of implantation. The injury gap of the mouse was 3 mm. A biocompatible conduit cellular nerve graft was evolved using bioprinting technology and was implanted in BMSCs. BMSC and SC stem cells were used and applied to produce cellular cylinders encircled by collagen tubes. A nerve graft was inserted in a rat model for 40 weeks, and axon regrowth was measured.^289^ Zhu et al. reported damage repairing by fabrication of nanobioink. The SLA‐assisted 3D bioprinting method developed this neural scaffold printing consisting of stem cells, graphene nanoplatelets, and GelMA. A higher concentration of the formulated hydrogel exhibited a high compressive modulus. A suitable stem cell growth environment was created due to the excellent cell viability and biocompatibility of GelMa.^223^

#### 7.2.4 Skeletal muscle tissue

Researchers construct skeletal muscle tissues by utilizing 3D bioprinting methods. Costantini et al. reported skeletal muscle tissues evolved from photocurable PEG‐fibrinogen copolymer encapsulating C2C12 cells.^290^ A high degree of alignment of C2C12 cells in the direction of PEG‐fibrinogen biopolymer displayed higher sarcomerogenesis. Implantation of developed hydrogel into SIDC mice demonstrated the regeneration of muscle tissues and myotube development after 28 days of administration. Layer‐by‐layer SLA method was applied for artificial muscle construction. A photoactive commercially available material was presented with a resolution of as high as 37 μm in the horizontal plane. The measurement of tensile testing, stress–strain behavior, and rheological studies showed large actuator amplitudes of the evolved device.^291^ Choi et al. and coworkers fabricated bioink using decellularized skeletal muscle ECM (mdECM) to regenerate skeletal muscle using 3D cell‐printing technology.^292^ Volumetric muscle loss (VML) is a type of irrecoverable muscle injury that occurs when muscle loss is higher than 20% is called VML. Using 3D cell‐printing technology decellularized ECM (dECM) was fabricated and applied for VML treatment.^293^ A bioengineered, implantable skeletal muscle tissue made from human primary muscle progenitor cells (hMPCS) using the 3D bioprinting approach. The fabricated tissue showed a high‐order multilayered muscle bunch made by aligned, closely packed myofiber‐like formation, which also offers 82% functional recovery in a tibialis anterior muscle defect after 8 weeks of implantation.^294^

#### 7.2.5 Bone and cartilage

Another promising field of 3D bioprinting application is cartilage reconstruction and bone regeneration. Based on injecting bioprinting technology, Cui et al. fabricated human chondrocytes entrapped PEG–DMA hydrogel for cartilage repair.^295^ The compressive modulus of the device was similar to the articular cartilage properties of humans. Hand‐operated PEG–DMA hydrogel shows cell viability of only 72%, whereas 3D bioprinted hydrogel shows cell viability of 92%. The printed gel was firmly attached to the original tissue after sectioning. Markstedt et al. prepared a bioink consolidated nanofibrillated cellulose (NFC) and alginate crosslinker for cartilage tissue regeneration using extrusion‐based bioprinting technology. Chondrocytes stem cells were evenly spread in the nanocellulose‐based hydrogel matrix and showed a high cell viability of ∼84% after one week of culture. Rheological studies indicate good printability of the bioink at room temperature.^164^ The two main components for the regeneration of bone tissue are suitable cell types and correct biomaterials. Gao et al. reported a system for bone tissue engineering involving PEG‐DMA hydrogel, HA and bioactive glass that can be bioprinted for bone tissue construction.^295^ Human MSCs (hMSCs) were bioprinted in the hydrogel. The developed system shows maximum cell viability of 92% and a compressive modulus of 405 kPa after 21 days of culture. Holmes et al. demonstrated a 3D nano and microvascular scaffold for bone reconstruction.^262^ The PLA‐based platforms were chemically modified by nanohydroxyapatite showing a good vascular flow profile and bone‐like effects. This study showed excellent cell adhesion of hMSC, osteogenic differentiation and cell proliferation, vascular cell growth, and emigration with HUVECs.

### 7.3 Clinical researches in 3D bioprinting

The additive manufacturing process of 3D bioprinting has offered a feasible solution in facing the various threats and challenges faced by the occurrence of new pathogens and several diseases associated with them. It has helped face the increasing demand for the new therapeutic method and the increase in drug discovery. This technology has been acting like a boon to the medical community due to its wide use for treating diseases like cancer, cardiovascular disease, central and peripheral nervous system diseases, diabetes, and so on.^296^ The 3D bioprinting technology is being widely used to develop several models to study cancer, especially metastasis and invasion studies. The 3D models were used to understand the antagonism of cancer cells toward the chemotherapeutic drugs and bone models for cancer metastasis. The laser printing technique was used to fabricate 3D cellular models depicting the exact physiological functions of cells.^237^, ^297^

This technique was further used to construct cancer models to study the cancer beginning and development on bioprinted pancreatic cell and spheroid models. 3D printing polymers like PLA and ABS are used with 0.75–1 mm slice thickness. Real functional cardiovascular models are fabricated using 3D printing materials including TangoPlus and Verowhite and mock circulatory systems.^298^, ^299^ Neural stem cell‐laden PU hydrogels were studied, and significant healing was observed to the injured CNS in the zebrafish embryo neural injury model.^300^ Lung models were infected with H1N1 and H3N2 influenza strains to study the protein expression and immunological activity. Further, these models can be used to develop antiviral compounds for studying viral replication. Several studies for designing models to study the replication of the SARS‐Cov2 virus are underway.^301^, ^302^ Various studies show promising results but are still not ready for clinical use. Albanna et al. developed a proof‐of‐concept skin bioprinter, as well as Dvir and his team also developed autologous cardiac patches, which are yet to be ready for clinical use. Various 3D organoids are under clinical trials, such as TUMOVASC (NCT04826913) and GLIOMANOID (NCT03971812). Several clinical trials like NCT037351 have been found in dentistry and orthopedic surgery for the safety and efficiency of dentures, implants and orthotic devices (Table 5).^303^, ^304^, ^305^

**TABLE 5:** Clinical studies based on 3D printing technique^303^

SL. No.	Interventions/procedures	Phases	Application and sponsor	NCT Number
1.	3D printing	I(Recruiting)	Breast cancer and reconstruction, Xijing Hospital	NCT03348293
2.	Thrombus aspiration	NA(Recruiting)	STEMI‐ST‐Elevation Myocardial infarction; Thrombi; MicroRNA, The Aristotle University of Thessaloniki; Hellenic Centre For Marine Research; Harvard Medical School; Centre for Research and Technology Hellas	NCT03832153
3.	Product: AuriNovo	I	Microtia,3D BioTherapeutics	NCT04399239
4.	Biological: Blood Samples; surgical tissue samples	NA(Recruiting)	Plastic surgeries, Assistance Publique Hopitaux De Marseille	NCT04925323
5.	PCL‐TCP Scaffold;Geistlich Bio‐Gide collagen membrane	NA	Bone resorption after tooth extraction, National Dental Centre (Singapore); Osteopore International	NCT03735199

## 8 CONCLUSION AND FUTURE PROSPECTS

3D bioprinting is an emerging technology growing daily by changing technological advancements in various fields. Its versatility makes it closer to those undisclosed and untreated medical problems, and the 3D construction is further essential to combat these problems in a skilled manner. This review provides a detailed description of all the parameters required for 3D printing. It has given an insight into various biomedical applications and how this technology has helped make the applications more accessible and efficient than previous models and studies. The technology has helped to fabricate artificial arteries for organs and is further used for in vivo and in vitro studies for several biomedical applications. Although the scale and functionality of 3D printed constructs are increasing daily, fabrication and printing of complex tissue constructs with greater physiological demand have been challenging. It considers the pivotal initial state of the printed object and assures its state‐of‐the‐art technology. To overcome the challenges, one needs further development toward scaling up the technology.

Deep learning has achieved remarkable attention due to increased computational power and availability of a massive amount of data. Though it is remarkably useful in domains such as computer vision and natural language, deep learning is comparatively lesser common in the application of 3D bioprinting. 3D bioprinting involves a series of operations where deep learning can be employed to streamline current workflow better and improve biological outcomes. Likewise, the computer is a branch of ML, which obeys hardcoded rules encoded by experts. ML helps optimize systems through more innovative and accurate use of products, materials, and services. In terms of 3D printing processes, ML can minimize cost, reduce fabrication time and improve the quality. It is a three‐way method supervised, unsupervised, and reinforcement learning. ML helps improve fabrication by indicating the process conditions and optimizing process parameters. To detect defects such as wrong‐positioned cells, curved layers, and microstructure errors in a fabrication process, ML can monitor the whole bioprinting process. For example, supporting cells in the tissue‐engineered scaffolds are complex and grow expectedly to achieve corresponding functions.

Final fabricated bioparts can be generated with better service if the accuracy is analyzed in advance. Still, we are away from the usefulness of deep learning in 3D bioprinting, and for the real perspective one needs a massive amount of data for predictions and optimization of the process. The key disadvantages of encapsulating living cells in biomaterials are the suspensions need to be preserved for an extended period in the material reservoir, the high fabrication time of organs and tissues reduces cell viability and restricts their bioactivity, an automated process required for loading and ejecting cell‐biomaterial suspension for scale‐up tissue and organ fabrication. Again, current 3D bioprinting is insufficient for fabricating a similar native vascular network because the size of the bioprinted tissues is larger than tens of micrometers. However, one need stiffness to preserve the entire structure for a prolonged time. However, a very high stiffness can impair cell viability. Isolation and incorporation of cells act as inhibiting factors in the efficiency of the printed 3D construct. Integration of advanced technology and efficient cellular integration will help achieve desired 3D constructs for biomedical applications with maximum efficiency, as only placing bioinks on the substrate is insufficient. 3D bioprinting has been efficiently and quickly evolving as a dominant tool for patterning living cells and biomaterials to create a 3D‐printed construct. However, this technology is still not able to achieve the results expected from the technology. 4D printing has emerged recently, a technology that integrates time with 3D bioprinting techniques. These printed objects can change their shape and functionality upon any external stimulus, cell fusion, or postprinting self‐assembly. The ability to vary with time has enabled fabricating of tissue structures that can undergo morphological changes. The resultant 4D bioprinted construct will help us to bring closer to the limitations and problems faced in the biomedical world. As a result, it can be a beneficial asset for solving several medical problems.

## AUTHOR CONTRIBUTION

Swikriti Tripathi conceived and prepared the structure of the manuscript. Subham Mandal drafted the initial manuscript. Sudepta Bauri revised and edited the manuscript. Pralay Maiti performed the conceptualization, supervision, editing, and resources for the work. All authors have read and approved the final manuscript.

## CONFLICT OF INTEREST

Author Pralay Maiti is an editorial board member of MedComm. Author Pralay Maiti was not involved in the journal's review or decisions related to this manuscript. The other authors declared no conflict of interest. All authors have read and approved the final manuscript.

## ETHICS STATEMENT

This review does not require an ethical statement.

## Data Availability

No new data were created or analyzed in this study, so data sharing is not applicable here.
